# Palbociclib plus letrozole as first-line therapy in estrogen receptor-positive/human epidermal growth factor receptor 2-negative advanced breast cancer with extended follow-up

**DOI:** 10.1007/s10549-018-05125-4

**Published:** 2019-01-10

**Authors:** H. S. Rugo, R. S. Finn, V. Diéras, J. Ettl, O. Lipatov, A. A. Joy, N. Harbeck, A. Castrellon, S. Iyer, D. R. Lu, A. Mori, E. R. Gauthier, C. Huang Bartlett, K. A. Gelmon, D. J. Slamon

**Affiliations:** 1 Department of Medicine (Hematology/Oncology), University of California San Francisco, Helen Diller Family Comprehensive Cancer Center, 1600 Divisadero St, 2nd Floor, San Francisco, CA 94115 USA; 2 Division of Hematology/Oncology, David Geffen School of Medicine at UCLA, Santa Monica, CA USA; 3 Department of Medical Oncology, Institut Curie, Paris, France; 4 Centre Eugène Marquis, Rennes, France; 5 Frauenklinik und Poliklinik Klinikum rechts der Isar, Technische Universität München, Munich, Germany; 6 Republican Clinical Oncology Dispensary, State Budget Medical Institution, Ufa, Russia; 7 Department of Oncology, Cross Cancer Institute, University of Alberta, Edmonton, AB Canada; 8 Department of Obstetrics and Gynecology, Brustzentrum der Universität München (LMU), Munich, Germany; 9 Breast Cancer Center, Memorial Cancer Institute, Hollywood, FL USA; 10 Patient and Health Impact, Pfizer Inc, New York, NY USA; 11 Clinical Statistics, Pfizer Inc, La Jolla, CA USA; 12 Global Product Development, Clinical, Pfizer S.r.l, Milan, Italy; 13 Global Product Development, Clinical, Pfizer Inc, San Francisco, CA USA; 14 Global Product Development, Clinical, Pfizer Inc, Collegeville, PA USA; 15 Department of Medical Oncology, British Columbia Cancer Agency, Vancouver, BC Canada

**Keywords:** Breast cancer, ER+, HER2−, Cyclin-dependent kinase inhibitor, Palbociclib, Letrozole

## Abstract

**Purpose:**

In the initial PALOMA-2 (NCT01740427) analysis with median follow-up of 23 months, palbociclib plus letrozole significantly prolonged progression-free survival (PFS) in women with estrogen receptor-positive (ER+)/human epidermal growth factor receptor 2-negative (HER2−) advanced breast cancer (ABC) [hazard ratio (HR) 0.58; *P* < 0.001]. Herein, we report results overall and by subgroups with extended follow-up.

**Methods:**

In this double-blind, phase 3 study, post-menopausal women with ER+/HER2− ABC who had not received prior systemic therapy for their advanced disease were randomized 2:1 to palbociclib-letrozole or placebo-letrozole. Endpoints include investigator-assessed PFS (primary), safety, and patient-reported outcomes (PROs).

**Results:**

After a median follow-up of approximately 38 months, median PFS was 27.6 months for palbociclib–letrozole (*n* = 444) and 14.5 months for placebo-letrozole (*n* = 222) (HR 0.563; 1-sided *P* < 0.0001). All subgroups benefited from palbociclib treatment. The improvement of PFS with palbociclib-letrozole was maintained in the next 2 subsequent lines of therapy and delayed the use of chemotherapy (40.4 vs. 29.9 months for palbociclib–letrozole vs. placebo-letrozole). Safety data were consistent with the known profile. Patients’ quality of life was maintained.

**Conclusions:**

With approximately 15 months of additional follow-up, palbociclib plus letrozole continued to demonstrate improved PFS compared with placebo plus letrozole in the overall population and across all patient subgroups, while the safety profile remained favorable and quality of life was maintained. These data confirm that palbociclib-letrozole should be considered the standard of care for first-line therapy in patients with ER+/HER2− ABC, including those with low disease burden or long disease-free interval. Sponsored by Pfizer; ClinicalTrials.gov: NCT01740427.

**Electronic supplementary material:**

The online version of this article (10.1007/s10549-018-05125-4) contains supplementary material, which is available to authorized users.

## Introduction

Endocrine therapy has been the primary first-line treatment for hormone receptor-positive (HR+)/human epidermal growth factor receptor 2-negative (HER2−) advanced breast cancer (ABC) [1–3]. Recently, guidelines have expanded to include the addition of a cyclin-dependent kinase 4/6 (CDK4/6) inhibitor in combination with endocrine therapy for the treatment of pre-menopausal/post-menopausal women with HR+/HER2‒ ABC [1–3].

In the PALOMA-2 study, palbociclib-letrozole significantly prolonged progression-free survival (PFS) versus placebo-letrozole [median PFS, 24.8 vs. 14.5 months, respectively; hazard ratio [HR], 0.576 (95% CI 0.463–0.718); *P* < 0.0001] [4, 5]. The primary analysis was conducted after a median 23 months of follow-up (data cut-off: February 26, 2016), with the investigators and patients remaining blinded to treatment assignments. Because patients with HR+/HER2− ABC receiving first-line therapy have diverse clinical and molecular presentations (e.g., de novo versus recurrent disease, visceral versus bone-only), response to endocrine-based therapy could be prolonged in a particular subgroup. Therefore, it is important to analyze the long-term efficacy of treatment with extended follow-up in different patient subgroups.

Currently, PALOMA-2 has the longest follow-up of any phase 3 study investigating CDK4/6 inhibitors for HR+/HER2− disease. In this report, we present updated efficacy, safety, and patient-reported outcome (PRO) results for the overall PALOMA-2 study population and across subgroups after extended patient follow-up. This study is ongoing to collect overall survival data.

## Methods

### Study design, treatment, and patient eligibility criteria

Eligibility criteria and study design details were reported previously [4]. PALOMA-2 was a double-blind, international, phase 3 study in which women with estrogen receptor-positive (ER+)/HER2− advanced breast cancer were randomized 2:1 to receive letrozole 2.5 mg/day continuously and either palbociclib (125 mg/day, 3 weeks on followed by 1 week off of a 4-week cycle) or matching placebo. The study protocol was reviewed and approved by institutional review boards/independent ethics committees at each site (Table S1) and was conducted in accordance with Good Clinical Practice principles and the Declaration of Helsinki. Informed consent was obtained from all individual participants included in the study.

### Endpoints and assessments

The study’s primary endpoint was investigator-assessed PFS, defined as the time from date of randomization to the date of first documented objective disease progression (per Response Evaluation Criteria in Solid Tumors, v1.1) or death due to any cause, whichever occurred first. A blinded, independent central review of all patients was performed in a third party facility. Secondary endpoints included patient-reported outcomes (PROs), pharmacokinetics, and safety assessments. Subgroup analyses by baseline characteristics were pre-specified. Patient-reported breast cancer-specific health-related quality of life (HRQOL) was assessed using the Functional Assessment of Cancer Therapy-Breast (FACT-B) completed on-site at baseline (day 1 of cycle 1), day 1 of cycles 2 and 3, and day 1 of every other cycle from cycle 5 until progression or end of treatment [6–8]. Adverse events (AEs) were recorded during study treatment until 28 days after the last treatment dose. AEs were graded for severity according to the National Cancer Institute Common Terminology Criteria for Adverse Events version 4.0 and classified according to Medical Dictionary for Regulatory Activities (MedDRA, v20.0).

In addition to these endpoints, time to initiation of subsequent anticancer therapies (including chemotherapy) was assessed in the overall study population as an exploratory analysis to investigate whether palbociclib–letrozole treatment affected subsequent therapies. The time to initiation of subsequent therapy was defined as the time from randomization to the start date of subsequent systemic anticancer therapy or death from any cause, whichever occurred first.

### Statistical analyses

Progression-free survival was evaluated in the overall intent-to-treat (ITT) population and in preplanned subgroups defined by their baseline characteristics. Time to subsequent therapies was evaluated in the ITT population. The Kaplan–Meier method was used to estimate median PFS and time to subsequent systemic anticancer therapies (including chemotherapy) by treatment arm. Hazard ratios for PFS and time to first and second subsequent systemic anticancer therapies were estimated from the Cox proportional hazards model with a 95% CI; 1-sided *P* values were from the log-rank test. No adjustments were made for multiple testing. Repeated-measures mixed effects models were used to assess the effect on changes from baseline in patient-reported quality of life using intercept term, treatment, time, treatment-by-time, and baseline as covariates. A subpopulation treatment effect pattern plot (STEPP) [9] analysis was performed to explore whether PFS benefit was affected by a patient’s treatment-free interval (TFI) at baseline, where TFI (equivalent to the protocol-defined disease-free interval [DFI]) was defined as the time from the end of (neo)adjuvant therapy to disease progression. AE data were analyzed for patients who received ≥ 1 dose of study drug.

## Results

### Patients

From February 2013 through July 2014, 666 post-menopausal women were enrolled and randomly assigned to receive palbociclib-letrozole (*n* = 444) or placebo-letrozole (*n* = 222) (Fig. S1). Demographics and baseline disease characteristics were well balanced between treatment arms and similar to that previously reported [4] (Table S2). Exposure to palbociclib or placebo is summarized in Table S3.

### Efficacy

**Fig. 1:**
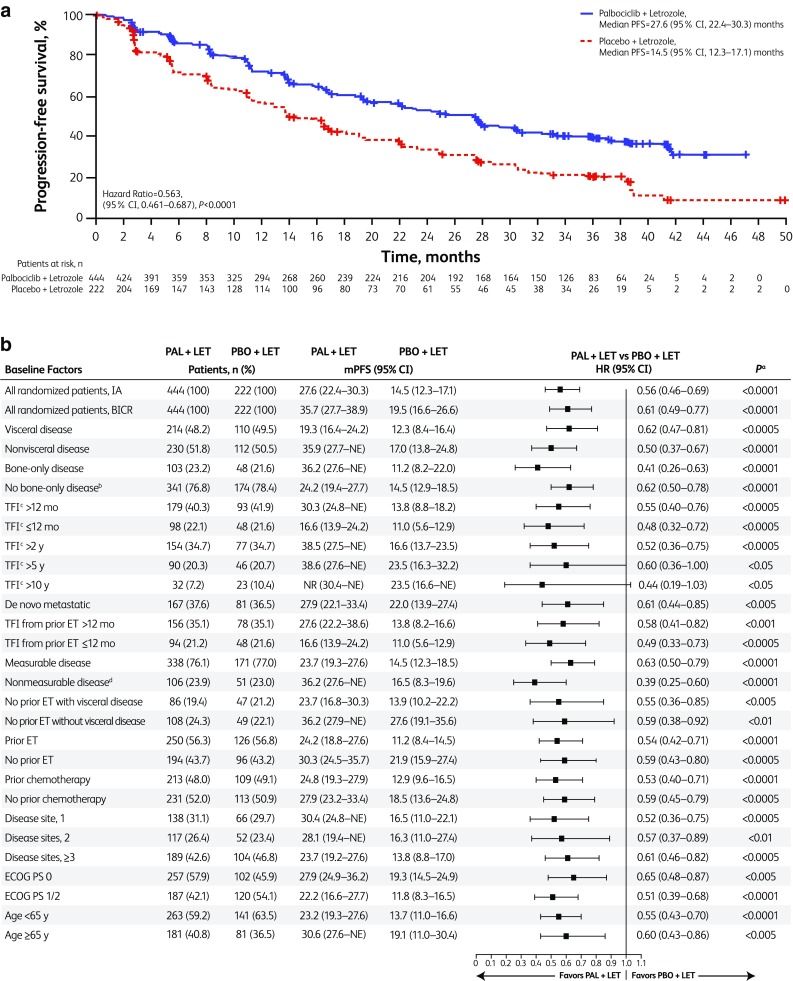
**a** Investigator-assessed progression-free survival (ITT population). **b** Forest plot of investigator-assessed PFS overall and across subgroups (ITT population). *BICR* blinded independent central review, *ECOG PS* Eastern Cooperative Oncology Group performance status, *ET* endocrine therapy, *HR* hazard ratio, *IA* investigator assessed, *ITT* intent-to-treat, *LET* letrozole, *PFS* progression-free survival, *NE* not estimable, *NR* not reached, *PAL* palbociclib, *PBO* placebo, *TFI* treatment-free interval. a: 1-sided *P* value from the log-rank test. b: Per tumor site. c: Protocol-defined disease-free interval is equivalent to TFI in this analysis and refers to TFI since completion of prior (neo)adjuvant therapy and onset of metastatic disease or disease recurrence. d: A few patients initially enrolled as having measurable disease were later found to have non-measurable disease beyond bone-only disease

At the new data cut-off date (May 31, 2017), median (interquartile range) follow-up was 37.6 (37.2–38.0) months in the palbociclib-letrozole arm and 37.3 (36.3–37.9) months in the placebo-letrozole arm. Investigator-assessed PFS was significantly longer with palbociclib-letrozole versus placebo-letrozole in the ITT population, with a median of 27.6 months (95% CI 22.4‒30.3) versus 14.5 months (12.3‒17.1), respectively [HR, 0.563 (95% CI 0.461–0.687); *P* < 0.0001] (Fig. 1a). This improvement in PFS with palbociclib was supported by the results of the updated blinded independent central review: median PFS 35.7 months (95% CI 27.7–38.9) versus 19.5 months (16.6–26.6), respectively [HR, 0.611 (95% CI 0.485–0.769); *P* < 0.0001].

**Fig. 2:**
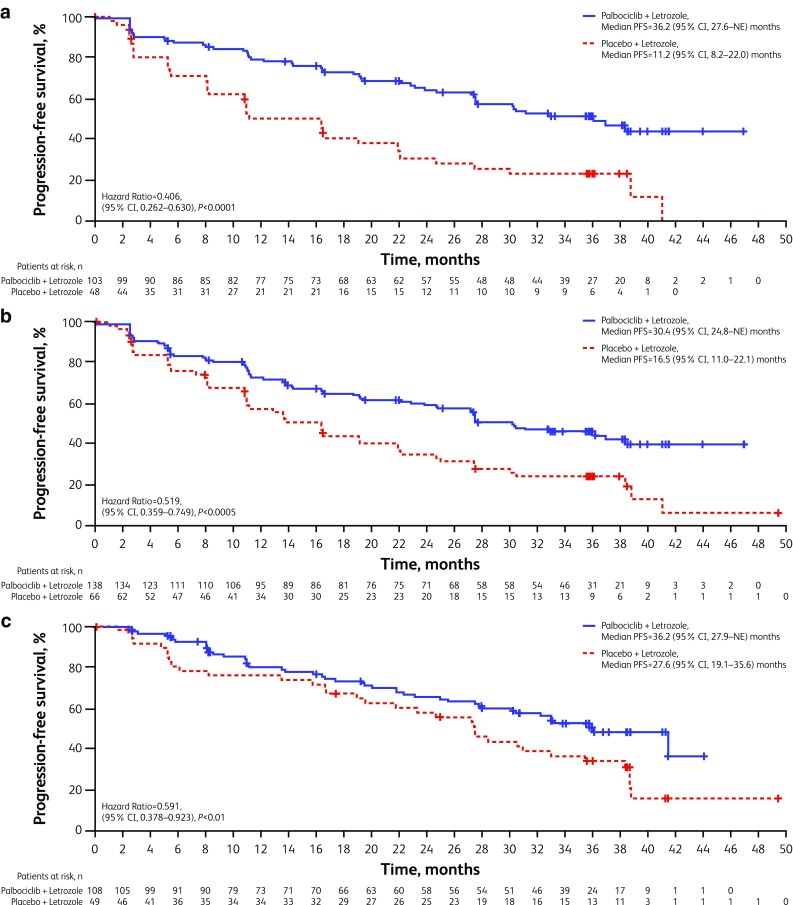
Investigator-assessed PFS in subgroups of patients (ITT population). Kaplan–Meier curves for **a** bone-only and **b** single disease site—both representing low disease burden—and **c** no prior endocrine therapy with non-visceral disease. *HR* hazard ratio, *ITT* intent-to-treat, *NE* not estimable, *PFS* progression-free survival

Median PFS was also longer with palbociclib-letrozole across all subgroups examined (Fig. 1b). Notably, a substantial benefit in PFS with palbociclib-letrozole was observed for patients with a low disease burden such as non-measurable disease (4% visceral), bone-only disease, or single disease site. For patients with non-visceral disease who did not receive prior endocrine therapy, the median PFS exceeded 3 years with palbociclib-letrozole (Figs. 1b, 2). In addition, the magnitude of the PFS benefit from palbociclib-letrozole versus placebo-letrozole was consistent, regardless of baseline TFI or whether patients had received prior endocrine therapy (Figs. S2 and 1b).

### Subpopulation treatment effect pattern plot analysis

To better understand whether the PFS treatment effect is influenced by baseline TFI, we performed a STEPP analysis in patients who had received (neo)adjuvant endocrine therapy. The treatment effect of PFS was generally consistent regardless of TFI (Fig. S3). No TFI cut-off changed the clinical benefit (Fig. S2).

### Time to subsequent anticancer therapies

**Fig. 3:**
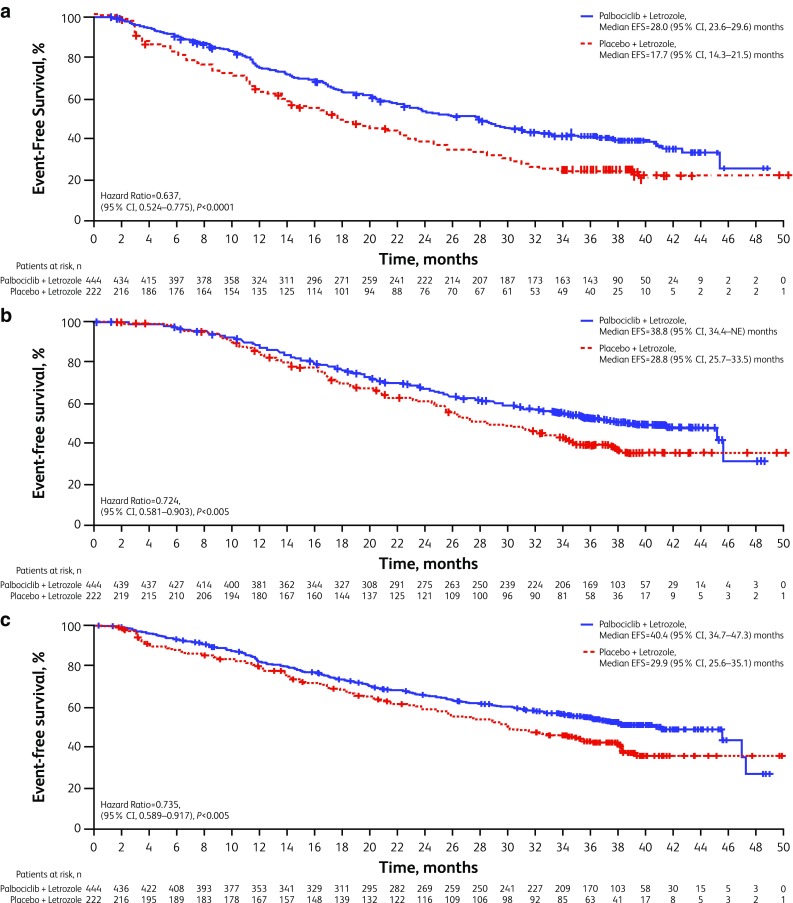
Kaplan–Meier estimates of time to initiation of subsequent systemic anticancer therapies (anticancer treatment included any anticancer related systemic therapy and surgery for the disease under study) (ITT population) **a** Time from randomization to first subsequent therapy. **b** Time from randomization to second subsequent therapy (if the difference in time to initiation of the second subsequent therapy between the 2 treatment arms was shortened compared with the difference between the median PFS values, it may suggest that the treatment benefit of the first subsequent therapy was compromised. If the difference was similar, it suggests no compromise regarding the efficacy of the first subsequent therapy). **c** Time from randomization to first subsequent chemotherapy. *EFS* event-free survival, *ITT* intent-to-treat

To explore whether the combination therapy had a potential impact on subsequent line therapies after permanent palbociclib discontinuation, analyses of time to initiation of first and second subsequent therapies were conducted. Median time from randomization to the initiation of the first subsequent therapy was 28.0 (95% CI 23.6–29.6) versus 17.7 (14.3–21.5) months with palbociclib–letrozole versus placebo-letrozole (Fig. 3a). The second subsequent systemic anticancer therapy was also significantly delayed in the palbociclib-letrozole arm compared with the placebo-letrozole arm at 38.8 (95% CI, 34.4‒not estimable) months versus 28.8 (25.7‒33.5) months, respectively (Fig. 3b). In both analyses, the 10 month difference in PFS benefit from palbociclib observed in the primary PFS analysis was preserved, suggesting that the treatment benefit of the first subsequent therapy was not compromised by palbociclib. The median time to first-line subsequent chemotherapy was 40.4 (34.7‒47.3) months versus 29.9 months (25.6‒35.1) for patients treated with palbociclib-letrozole versus placebo-letrozole (Fig. 3c).

### Types of first subsequent therapy

Among 227 palbociclib–letrozole patients and 150 placebo–letrozole patients who received subsequent systemic anticancer therapies after permanent study treatment discontinuation, endocrine therapy was the most common first subsequent treatment in patients from both arms (60.8% and 58.0%, respectively), followed by chemotherapy (36.6% and 34.0%; Table S4). Some patients received a second subsequent therapy; common second therapies are listed in Table S5.

### Safety

With the additional 15 months of follow-up, no new safety signals were observed for palbociclib-letrozole. Over the entire study period, permanent discontinuation because of all-causality treatment-emergent AEs occurred in 54 (12.2%) patients in the palbociclib arm and 13 (5.9%) in the placebo arm. Neutropenia was the most frequently reported any-grade AE with palbociclib-letrozole (81.8% vs 6.3% with placebo-letrozole) (Table S6). Most events in the palbociclib-letrozole arm were of grade 3 severity (57.4%); however, neutropenia rarely led to permanent study discontinuation (*n* = 8 [1.8%]), and febrile neutropenia was rare (*n* = 9 [2.0%]). Treatment-emergent serious AEs (SAEs) of any cause occurred in 23.6% of palbociclib-letrozole patients and 15.3% of placebo-letrozole patients. Infections were the most commonly reported SAE in both arms (5.2% and 4.1%, respectively).

### Patient-reported outcomes

**Fig. 4:**
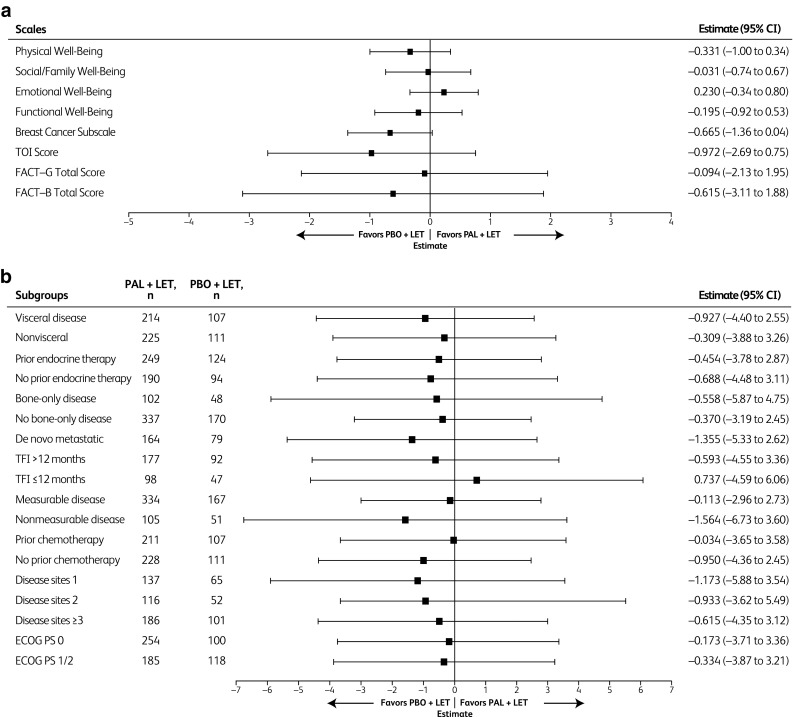
Between-treatment comparison of changes from baseline for FACT-B scores (PRO analysis set included patients in the PRO-evaluable population [i.e., patients with a baseline and ≥ 1 postbaseline assessment before the end of the study treatment]) **a** FACT-B scales of overall scores. **b** FACT-B total score by subgroups. *BC* breast cancer, *ECOG PS* Eastern Cooperative Oncology Group performance status, *FACT-B* Functional Assessment of Cancer Therapy-Breast, *FACT-G* Functional Assessment of Cancer Therapy-General, *LET* letrozole, *PAL* palbociclib, *PBO* placebo, *PRO* patient-reported outcome, *TFI* treatment-free interval, *TOI* Trial Outcome Index

Patient-reported HRQOL as assessed by the FACT-B total score was maintained with palbociclib–letrozole. The overall change from baseline in FACT-B total scores was not significantly different (*P* = 0.629) between the palbociclib-letrozole and placebo-letrozole arms. No statistically significant differences were observed between the treatment arms in change from baseline scores for any of the subscales assessed (Fig. 4a). The results for patient-reported HRQOL were consistent across all subgroups, including patients with bone-only disease and long TFI (Fig. 4b).

## Discussion

Single-agent sequential endocrine therapy, which is associated with less drug toxicity than chemotherapy, has been the recommended standard of care for HR+/HER2− ABC in the first-line setting [1–3]; however, resistance to single-agent endocrine therapy and relapse over time are inevitable. As such, further research is warranted on the inclusion of targeted agents in endocrine-based therapy to delay resistance and prolong the window of endocrine sensitivity. Following the positive results from the PALOMA-1, -2, and -3 studies [4, 10, 11], MONALEESA-2, -3, and -7 [12, 13], and the MONARCH 2 and 3 studies [14, 15], the international treatment guidelines now include recommendations for the use of CDK4/6 inhibitors in combination with endocrine therapy for the treatment of pre-menopausal and post-menopausal women with HR+/HER2‒ ABC as first-line standard therapy [1–3].

To date, PALOMA-2 offers the longest follow-up of any phase 3 study evaluating a CDK4/6 inhibitor in patients with ABC and no prior systemic treatment for their advanced disease. After 37.6 months of follow-up, palbociclib-letrozole consistently improved median PFS compared with placebo-letrozole in the overall population and across all subgroups of patients with ER+/HER2‒ ABC. Of note, patients with a low disease burden or a demonstrated sensitivity to endocrine monotherapy derived substantial PFS benefit from the addition of palbociclib to letrozole (> 3 years median PFS); these findings were confirmed by a STEPP analysis of TFI. The PFS benefit for patients with a low disease burden receiving palbociclib-letrozole should also be viewed in the context of results from another analysis of the PALOMA-2 population which concluded that patients without progression versus those who progressed showed a significantly greater delay in deterioration of HRQOL [16].

The role of abemaciclib plus letrozole or anastrozole as initial treatment for HR+/HER2‒ ABC is being investigated in the MONARCH 3 trial [15]. In contrast to the PALOMA-2 study in which all subgroups of patients benefited from the addition of palbociclib to letrozole, an exploratory subgroup analysis from MONARCH 3 suggested that patients with a better prognosis at baseline (i.e., > 36 months TFI or bone-only disease) derived no further benefit from the addition of abemaciclib to endocrine therapy. However, comparisons across studies can be confounding when the duration of follow-up is too short for accrual of events in patients with endocrine-sensitive disease or bone-only disease.

The updated PALOMA-2 results for the subgroup of patients with non-visceral disease who had not received previous endocrine therapy can also be viewed alongside the Fulvestrant and Anastrozole Compared in Hormonal Therapy Naive Advanced Breast Cancer (FALCON) trial results [17]. In a pre-specified subgroup of 208 women who had not received prior endocrine therapy and who did not have visceral disease, the median PFS was 22.3 versus 13.8 months with fulvestrant versus anastrozole, respectively, HR 0.59 (95% CI 0.42–0.84) [17]. Although cross-study comparisons are inexact, in PALOMA-2, the median PFS was 36.2 months (Fig. S3c) for women with non-visceral disease and no prior endocrine therapy who received palbociclib and letrozole, as compared to the 22.3 months PFS seen with fulvestrant in patients with non-visceral disease in the FALCON study. Among the patients who had not received prior endocrine therapy or had non-visceral disease in the PALOMA-2 study, those who received palbociclib-letrozole for > 3 years maintained a quality of life not significantly different from patients receiving placebo-letrozole therapy (Fig. 4); furthermore, this quality of life is similar to that of a normal healthy population.

The results for median time to initiation of the first and second subsequent systemic therapy in this study suggest that the treatment benefit of the first subsequent therapy was not compromised by palbociclib. Additional clinical studies are needed to confirm these findings.

Similarly, palbociclib plus letrozole therapy delayed the initiation of first subsequent chemotherapy. More than one-third of patients in this study received chemotherapy as their first subsequent line of therapy after disease progression on the study drug, which could suggest theirs was a higher-risk disease. The longer the initiation of salvage chemotherapy can be postponed, the longer patients can be spared from increased toxicities associated with these drugs, which may have a more negative effect on quality of life than less toxic agents [18] and, more importantly, have limited efficacy after endocrine therapy failure. Thus, the observed prolonged time to initiation of first subsequent chemotherapy following palbociclib is of clinical relevance to patients with ER+/HER2− ABC because it postponed the onset of endocrine resistance and may offer a therapeutic advantage in a setting with unmet medical needs [18].

Of the patients who progressed on palbociclib–letrozole in the first-line setting, 70 (30.8%) and 49 (21.6%) in the next immediate line of therapy switched to fulvestrant and exemestane therapy, respectively. These data suggest that challenging patients with single-agent endocrine therapy is feasible following progression on CDK inhibitor therapy.

None of the phase 3 studies of CDK4/6 inhibitors as first-line treatment have reported overall survival. Due to the chronic and prolonged indolent nature of HR+ MBC, PALOMA-2 has not yet reached the prerequisite number of events to trigger overall survival analysis. In the absence of overall survival data, the median PFS durations for subgroups of patients in the first and immediate subsequent line of therapy post-progression are of interest. Our analysis showed that the time to subsequent line was prolonged by the addition of palbociclib over endocrine therapy alone and treatment effect was maintained, which provides early evidence for the long-term impact on patient outcomes.

## Conclusions

In this study, after approximately 15 additional months of follow-up, palbociclib-letrozole consistently improved PFS across all clinically relevant subgroups and substantially delayed the next line of therapy without decreasing its duration of use. Furthermore, the safety profile of the combination remained consistent with previous observations [4]. The PROs confirm that quality of life was maintained in the overall population and across subgroups. Collectively, these data reinforce that palbociclib-letrozole should be regarded as an important first-line therapy option for patients with HR+/HER2− ABC.

## Electronic supplementary material

Below is the link to the electronic supplementary material.

Supplementary material 1 (PDF 662 KB)

## Data Availability

Upon request, and subject to certain criteria, conditions and exceptions (see https://www.pfizer.com/science/clinical-trials/trial-data-and-results for more information), Pfizer will provide access to individual de-identified participant data from Pfizer-sponsored global interventional clinical studies conducted for medicines, vaccines and medical devices (1) for indications that have been approved in the US and/or EU or (2) in programs that have been terminated (i.e., development for all indications has been discontinued). Pfizer will also consider requests for the protocol, data dictionary, and statistical analysis plan. Data may be requested from Pfizer trials 24 months after study completion. The de-identified participant data will be made available to researchers whose proposals meet the research criteria and other conditions, and for which an exception does not apply, via a secure portal. To gain access, data requestors must enter into a data access agreement with Pfizer.

## Abbreviations

ABC: Advanced breast cancer
AE: Adverse event
BC: Breast cancer
BICR: Blinded independent central review
CDK4/6: Cyclin-dependent kinase 4/6
DFI: Disease-free interval
ECOG PS: Eastern Cooperative Oncology Group performance status
EFS: Event-free survival
ET: Endocrine therapy
FACT-B: Functional Assessment of Cancer Therapy-Breast
FACT-G: Functional Assessment of Cancer Therapy-General
FALCON: Fulvestrant and Anastrozole Compared in Hormonal Therapy Naive Advanced Breast Cancer
HER2‒: Human epidermal growth factor receptor 2-negative
HR: Hazard ratio
HR+: Hormone receptor-positive
HRQOL: Health-related quality of life
IA: Investigator assessed
ITT: Intent-to-treat
LET: Letrozole
MedDRA: Medical Dictionary for Regulatory Activities
NE: Not estimable
NR: Not reached
PAL: Palbociclib
PBO: Placebo
PFS: Progression-free survival
PRO: Patient-reported outcome
SAE: Serious adverse event
STEPP: Subpopulation treatment effect pattern plot
TFI: Treatment-free interval
TOI: Trial Outcome Index
