# Supplementary material for: Palbociclib plus letrozole as first-line therapy in estrogen receptor-positive/human epidermal growth factor receptor 2-negative advanced breast cancer with extended follow-up
Source: Breast Cancer Res Treat. 2019 Jan 10;174(3):719–29. doi: 10.1007/s10549-018-05125-4 (PMC6438948; doi:10.1007/s10549-018-05125-4)
Supplement: Supplementary file 1 — Supplementary material 1 (PDF 662 KB) [file 10549_2018_5125_MOESM1_ESM.pdf]

Palbociclib plus letrozole as first-line therapy in estrogen receptor–positive/human epidermal growth factor receptor 2–negative advanced breast cancer with extended follow-up

Journal Name: *Breast Cancer Research and Treatment*

H. S. Rugo,[1]* R. S. Finn,[2] V. Diéras,[3,4] J. Ettl,[5] O. Lipatov,[6] A. A. Joy,[7] N. Harbeck,[8] A. Castrellon,[9] S. Iyer,[10] D. R. Lu,[11] A. Mori,[12] E. R. Gauthier,[13] C. Huang Bartlett,[14] K. A. Gelmon,[15] D. J. Slamon[2]

Address correspondence to:

Dr. Hope S. Rugo

Department of Medicine (Hematology/Oncology)

University of California San Francisco

Helen Diller Family Comprehensive Cancer Center

1600 Divisadero St, 2nd Floor

San Francisco, CA 94115 USA

Email: hope.rugo@ucsf.edu

Appendix: Supplementary Material

Table S1. List of Independent Ethics Committees or Institutional Review Boards

AUSTRALIA

Study Site Number	Independent Ethics Committee or Institutional Review Board Address(es)
1150	Hunter New England Human Research Ethics Committee<br>Locked Bag No. 1<br>New Lambton, NSW 2305<br>AUSTRALIA
1151	Hunter New England Human Research Ethics Committee<br>Locked Bag No. 1<br>New Lambton, NSW 2305<br>AUSTRALIA
1152	Hunter New England Human Research Ethics Committee<br>Locked Bag No. 1<br>New Lambton, NSW 2305<br>AUSTRALIA
1153	Hunter New England Human Research Ethics Committee<br>Locked Bag No. 1<br>New Lambton, NSW 2305<br>AUSTRALIA
1154	Epworth HealthCare Research and Ethics Committee<br>89 Bridge Road, Epworth Healthcare<br>Richmond, VIC 3121<br>AUSTRALIA
1155	Hunter New England Human Research Ethics Committee<br>Locked Bag No. 1<br>New Lambton, NSW 2305<br>AUSTRALIA

Study Site Number	Independent Ethics Committee or Institutional Review Board Address(es)
1156	Hunter New England Human Research Ethics Committee<br>Locked Bag No. 1<br>New Lambton, NSW 2305<br>AUSTRALIA
1157	Bellberry Human Research Ethics Committee<br>129 Glen Osmond Road<br>Eastwood, SOUTH AUSTRALIA 5063<br>AUSTRALIA
1159	Hunter New England Human Research Ethics Committee<br>Locked Bag No. 1<br>New Lambton, NSW 2305<br>AUSTRALIA
1160	Hunter New England Human Research Ethics Committee<br>Locked Bag No. 1<br>New Lambton, NSW 2305<br>AUSTRALIA
1164	Hunter New England Human Research Ethics Committee<br>Hunter New England Research Ethics and Governance Unit<br>Locked Bag 1<br>New Lambton, NSW 2305<br>AUSTRALIA
1166	Hunter New England Human Research Ethics Committee<br>Locked Bag No. 1<br>New Lambton, NSW 2305<br>AUSTRALIA
1200	Research Ethics Committee, Royal Adelaide Hospital<br>Level 3, Hansen Institute, IMVS Building, Royal Adelaide Hospital, North Terrace<br>Adelaide, SA 5000<br>AUSTRALIA

Study Site Number	Independent Ethics Committee or Institutional Review Board Address(es)
1279	Royal Perth Hospital Human Research Ethics Committee<br>Royal Perth Hospital, Ethics Office, Level 5 Colonial House<br>Perth, WA 6001<br>AUSTRALIA

BELGIUM

Study Site Number	Independent Ethics Committee or Institutional Review Board Address(es)
1036	Commissie Medische Ethiek van Universitaire Ziekenhuizen KU Leuven<br>Campus Gasthuisberg, Herestraat 49<br>Leuven, 3000<br>BELGIUM
1039	Commissie Medische Ethiek van de Universitaire Ziekenhuis K.U. Leuven<br>Campus Gasthuisberg E330, Herestraat 49<br>Leuven, 3000<br>BELGIUM
1054	COMMISSIE MEDISCHE ETHIEK VAN DE UNIVERSITAIRE ZIEKENHUIZEN KU LEUVEN<br>Campus Gasthuisberg, Herestraat 49<br>Leuven, B-3000<br>BELGIUM<br><br>Comite Ethique CHU Sart Tilman<br>1, avenue de l'hopital/Domaine universitaire B35, Tour 2, niveau -2E<br>Liege, 4000<br>BELGIUM
1076	Commissie Medische Ethiek van Universitaire Ziekenhuizen KU Leuven<br>Campus Gasthuisberg, Herestraat 49<br>Leuven<br>BELGIUM
1113	COMMISSIE MEDISCHE ETHIEK VAN DE UNIVERSITAIRE ZIEKENHUIZEN KU LEUVEN<br>Campus Gasthuisberg, Herestraat 49<br>Leuven, B-3000<br>BELGIUM
1142	Commissie Medische Ethiek van Universitaire Ziekenhuizen KU Leuven,<br>Campus Gasthuisberg, Herestraat 49,<br>Leuven, 3000<br>BELGIUM

Study Site Number	Independent Ethics Committee or Institutional Review Board Address(es)
1192	Commissie Medische Ethiek van Universitaire Ziekenuizen KU Leuven<br>Campus Gasthuisberg, Herestraat 49<br>Leuven, 3000<br>BELGIUM
1262	Commissie Medische Ethiek van Universitaire Ziekenuizen KU Leuven<br>Campus Gasthuisberg, Herestraat 49<br>Leuven, 3000<br>BELGIUM

CANADA

Study Site Number	Independent Ethics Committee or Institutional Review Board Address(es)
1074	Comite d'ethique de la recherche et de l'Evaluation des Technologies de la Sante<br>5400 Gouin Boulevard West, Hopital du Sacre-Coeur<br>Montreal, QC H4J 1C5<br>CANADA
1205	Centre for Applied Ethics, McGill University Health Centre (MUHC)<br>2155 Guy Street, Room 223.09<br>Montreal, QC H3H 2R9<br>CANADA
1219	Comite d'ethique de la recherche du CHU de Quebec<br>10, rue de l'Espinay, Universite Laval<br>Quebec, QC G1L 3L5<br>CANADA
1220	Ontario Cancer Research Ethics Board<br>661 University Avenue, Ontario Cancer Research Ethics Board, MaRS Centre Suite 510<br>Toronto, ON M5G 0A3<br>CANADA
1225	Nova Scotia Health Authority Research Ethics Board<br>5790 University Avenue, Room 118<br>Halifax, NS B3H 1V7<br>CANADA
1226	Health Research Ethics Board of Alberta - Cancer Committee<br>10104-103 Avenue NW, 1500<br>Edmonton, AB T5J4A7<br>CANADA
1231	UBC BCCA Research Ethics Board (BCCA REB)<br>Fairmont Medical Building, BC Cancer Agency, 750 West Broadway, Suite 902<br>Vancouver, BC V5Z 1H5<br>CANADA

Study Site Number	Independent Ethics Committee or Institutional Review Board Address(es)
1232	Ontario Cancer Research Ethics Board<br>MaRS Centre<br>c/o Ontario Institute for Cancer Research, 661 University Avenue, Suite 510<br>Toronto, ON M5G 0A3<br>CANADA
1233	Ontario Cancer Research Ethics Board<br>MaRS Centre, South Tower<br>C/o Ontario Institute for Cancer Research, 101 College Street, Suite 800<br>Toronto, ON M5G 0A3<br>CANADA
1239	Ontario Cancer Research Ethics Board (OCREB)<br>661 University Avenue, MaRS Centre - Suite 510<br>Toronto, ON M5G 0A3<br>CANADA
1240	Ontario Cancer Research Ethics Board<br>MaRS Centre, South Tower<br>C/o Ontario Institute for Cancer Research, 101 College Street, Suite 800<br>Toronto, ON M5G 0A3<br>CANADA
1241	University of Saskatchewan BioMedical Research Ethics Board (Bio-REB)<br>1607 110 Gymnasium Place, Research Ethics Office, University of Saskatchewan, NRC, Plant Biotechnology Research Institute<br>Saskatoon, SK S7N 4J8<br>CANADA
1273	Ontario Cancer Research Ethics Board<br>MaRS Centre, South Tower<br>101 College Street, Suite 800<br>Toronto, ON M5G 0A3<br>CANADA
1282	UBC BCCA Research Ethics Board (BCCA REB)<br>Fairmont Medical Building, BC Cancer Agency, 750 West Broadway, Suite 902<br>Vancouver, BC V5Z 1H5<br>CANADA

Study Site Number	Independent Ethics Committee or Institutional Review Board Address(es)
1291	Health Research Ethics Board of Alberta - Cancer Committee<br>10104-103 Ave NWC/O Alberta Innovates - Health Solutions, 1500<br>Edmonton, AB T5J 4A7<br>CANADA

CZECH REPUBLIC

Study Site Number	Independent Ethics Committee or Institutional Review Board Address(es)
1097	Eticka komise FN Olomouc<br>I.P. Pavlova 6<br>Olomouc, 775 20<br>CZECH REPUBLIC
	Eticka komise pro multicentricke klinicke hodnoceni FN Motol<br>Fakultni nemocnice Motol, V uvalu 84<br>Praha 5, 15006<br>CZECH REPUBLIC
1288	Eticka komise pro multicentricke klinicke hodnoceni FN Motol<br>Fakultni nemocnice Motol, V uvalu 84<br>Praha 5, 15006<br>CZECH REPUBLIC
	Eticka komise<br>Sokolska 581, FN Hradec Kralove<br>Hradec Kralove, 500 05<br>CZECH REPUBLIC

FRANCE

Study Site Number	Independent Ethics Committee or Institutional Review Board Address(es)
1037	CPP Ile de France 3<br>89 rue d'Assas, Hopital Tarnier<br>Paris, 75006<br>FRANCE
1062	CPP Ile de France 3<br>Hôpital Tarnier, 89, rue d’Assas<br>PARIS, 75006<br>FRANCE
1063	CPP Ile de France 3<br>Hôpital Tarnier, 89, rue d’Assas<br>PARIS, 75006<br>FRANCE
1064	CPP Ile de France 3<br>Hôpital TARNIER, 89, rue d’Assas<br>PARIS, 75006<br>FRANCE
1065	CPP Ile de France 3<br>Hôpital TARNIER, 89, rue d’Assas<br>PARIS, 75006<br>FRANCE
1066	CPP Ile de France 3<br>89 rue d'Assas, Hopital Tarnier<br>Paris, 75006<br>FRANCE
1182	CPP Ile de France 3<br>Hôpital Tarnier Cochin, 89, rue d’Assas<br>PARIS, 75006<br>FRANCE

Study Site Number	Independent Ethics Committee or Institutional Review Board Address(es)
1183	CPP Ile de France 3<br>Hôpital TARNIER, 89, rue d'Assas<br>PARIS, 75006<br>FRANCE
1285	CPP Ile de France 3<br>Hôpital Tarnier Cochin, 89, rue d'Assas<br>PARIS, 75006<br>FRANCE
1293	CPP Ile de France 3<br>Hôpital Tarnier, 89, rue d'Assas<br>PARIS, 75006<br>FRANCE
1296	CPP Ile de France 3<br>Hôpital Tarnier, 89, rue d'Assas<br>PARIS, 75006<br>FRANCE
1309	CPP Ile de France 3<br>Hôpital Tarnier Cochin, 89, rue d'Assas<br>PARIS, 75006<br>FRANCE
1317	CPP Ile de France 3<br>Hôpital Tarnier Cochin, 89, rue d'Assas<br>PARIS, 75006<br>FRANCE
1318	CPP Ile de France 3<br>Hôpital Tarnier, 89, rue d'Assas<br>PARIS, 75006<br>FRANCE

GERMANY

Study Site Number	Independent Ethics Committee or Institutional Review Board Address(es)
1041	Ethikkommission der Med. Fakultät der LMU München<br>Pettenkoferstr. 8a, Prof. Dr. Wolfgang Eisenmenger<br>80336 Munchen<br>GERMANY
1042	Ethikkommission der Med. Fakultät der LMU München<br>Pettenkoferstr. 8a, Prof. Dr. Wolfgang Eisenmenger<br>80336 Munchen<br>GERMANY
1045	Ethikkommission der Med. Fakultät der LMU München<br>Pettenkoferstr. 8a, Prof. Dr. Wolfgang Eisenmenger<br>80336 Munchen<br>GERMANY
1046	Ethikkommission der Med. Fakultät der LMU München<br>Pettenkoferstr. 8a, Prof. Dr. Wolfgang Eisenmenger<br>80336 Munchen<br>GERMANY
1119	Ethikkommission der Med. Fakultät der LMU München<br>Pettenkoferstr. 8a, Prof. Dr. Wolfgang Eisenmenger<br>80336 Munchen<br>GERMANY
1135	Ethikkommission der Med. Fakultät der LMU München<br>Pettenkoferstr. 8a, Prof. Dr. Wolfgang Eisenmenger<br>80336 Munchen<br>GERMANY
1136	Aerztekammer Nordrhein<br>Tersteegenstr. 31<br>Duesseldorf, 40474<br>GERMANY

Study Site Number	Independent Ethics Committee or Institutional Review Board Address(es)
	Ethikkommission der Med. Fakultät der LMU München<br>Pettenkoferstr. 8a, Prof. Dr. Wolfgang Eisenmenger<br>80336 Munchen<br>GERMANY
1137	Ethikkommission der Med. Fakultät der LMU München<br>Pettenkoferstr. 8a, Prof. Dr. Wolfgang Eisenmenger<br>80336 Munchen<br>GERMANY
1139	Ethikkommission der Med. Fakultät der LMU München<br>Pettenkoferstr. 8a, Prof. Dr. Wolfgang Eisenmenger<br>80336 Munchen<br>GERMANY
1184	Ethikkommission der Med. Fakultät der LMU München<br>Pettenkoferstr. 8a, Prof. Dr. Wolfgang Eisenmenger<br>80336 Munchen<br>GERMANY
1194	Ethikkommission der Med. Fakultät der LMU München<br>Pettenkoferstr. 8 IV, Zi. 1 4.07 bis I 4.10 (Hausanschrift), Postanschrift: Pettenkoferstr. 8a<br>Munich, 80336<br>GERMANY
1207	Ethikkommission der Med. Fakultät der LMU München<br>Pettenkoferstr. 8a, Prof. Dr. Wolfgang Eisenmenger<br>80336 Munchen<br>GERMANY
1209	Ethikkommission der Med. Fakultät der LMU München<br>Pettenkoferstr. 8a, Prof. Dr. Wolfgang Eisenmenger<br>80336 Munchen<br>GERMANY
1210	Ethikkommission der Med. Fakultät der LMU München<br>Pettenkoferstr. 8a, Prof. Dr. Wolfgang Eisenmenger<br>80336 Munchen<br>GERMANY

Study Site Number	Independent Ethics Committee or Institutional Review Board Address(es)
1211	Ethikkommission de Landesarztekammer Hessen<br>Im Vogelsgesang 3<br>Frankfurt, 60488<br>GERMANY
1229	Ethikkommission der Med. Fakultät der LMU München<br>Ethikkommission der Med. Fakultät der LMU München, Prof. Dr. Wolfgang Eisenmenger Pettenkoferstr. 8a<br>Munich, BAVARIA 80336<br>GERMANY
1302	Ethikkommission der Med. Fakultät der LMU München<br>Pettenkoferstr. 8a, Prof. Dr. Wolfgang Eisenmenger<br>80336 Munchen<br>GERMANY
1303	Ethikkommission der Med. Fakultaet der LMU Muenchen<br>Pettenkoferstr. 8a, Prof. Dr. Wolfgang Eisenmenger<br>Munich, 80336<br>GERMANY
1312	Ethikkommission der Med. Fakultät der LMU München<br>Pettenkoferstr. 8a, Prof. Dr. Wolfgang Eisenmenger<br>80336 Munchen<br>GERMANY

HUNGARY

Study Site Number	Independent Ethics Committee or Institutional Review Board Address(es)
1061	Egeszsegugyi Tudomanyos Tanacs<br>Zrinyi u.3<br>Budapest, 1051<br>HUNGARY
1100	Egeszsegugyi Tudomanyos Tanacs<br>Zrinyi u.3<br>Budapest, 1051<br>HUNGARY
1145	Egeszsegugyi Tudomanyos Tanacs Klinikai<br>Arany Janos u. 6-8., Farmakologiai Etikai Bizottsag<br>Budapest, 1051<br>HUNGARY
1227	Egeszsegugyi Tudomanyos Tanacs<br>Klinikai Farmakologiai Etikai Bizottsaga<br>Arany Janos u. 6-8.<br>Budapest, 1051<br>HUNGARY
1228	Egeszsegugyi Tudomanyos Tanacs<br>Klinikai Farmakologiai Etikai Bizottsaga<br>Arany Janos u. 6-8.<br>Budapest, 1051<br>HUNGARY
1284	Egeszsegugyi Tudomanyos Tanacs<br>Zrinyi u.3<br>Budapest, 1051<br>HUNGARY

IRELAND

Study Site Number	Independent Ethics Committee or Institutional Review Board Address(es)
1068	Clinical Research Ethics Committee of the Cork Teaching Hospitals<br>6 Little Hanover Street, Lancaster Hall<br>Cork<br>IRELAND
1069	Clinical Research Ethics Committee of the Cork Teaching Hospitals<br>6 Little Hanover Street, Lancaster Hall<br>Cork<br>IRELAND
	Mater Multi-Centre Clinical Trials Advisory Group<br>Eccles Street, Mater Misericordiae University Hospital<br>Dublin 7<br>IRELAND
1098	Clinical Research Ethics Committee of the Cork Teaching Hospitals<br>6 Little Hanover Street, Lancaster Hall<br>Cork<br>IRELAND
	Mater Multi-Centre Clinical Trials Advisory Group<br>Eccles Street, Mater Private Hospital<br>Dublin 7<br>IRELAND
1101	Clinical Research Ethics Committee of the Cork Teaching Hospitals<br>6 Little Hanover Street, Lancaster Hall<br>Cork<br>IRELAND
1102	Clinical Research Ethics Committee of the Cork Teaching Hospitals<br>6 Little Hanover Street, Lancaster Hall<br>Cork<br>IRELAND

Study Site Number	Independent Ethics Committee or Institutional Review Board Address(es)
1104	Clinical Research Ethics Committee of the Cork Teaching Hospitals<br>6 Little Hanover Street, Lancaster Hall<br>Cork<br>IRELAND
1141	The Clinical Research Ethics Committee of the Cork Teaching Hospitals<br>6 Little Hanover Street, Lancaster Hall<br>Cork<br>IRELAND
1247	Clinical Research Ethics Committee of the Cork Teaching Hospitals<br>6 Little Hanover Street, Lancaster Hall<br>Cork<br>IRELAND
1248	Clinical Research Ethics Committee of the Cork Teaching Hospitals<br>6 Little Hanover Street, Lancaster Hall<br>Cork<br>IRELAND

ITALY

Study Site Number	Independent Ethics Committee or Institutional Review Board Address(es)
1050	Comitato Etico dell'Azienda USL Frosinone<br>Via Armando Fabi<br>Frosinone, 03100<br>ITALY
1060	Comitato Etico<br>Via Ripamonti, 435, Istituto Europeo Oncologico<br>Milano, 20141<br>ITALY
1120	Ethics Committee of the Area Vasta Nord Ovest Autonomous<br>Section of the Regional Ethic Committee for Clinical Investigation<br>Via Roma 67<br>Pisa, 56126<br>ITALY
	Comitato Etico<br>Via Elio Chianesi, 53<br>Roma, 00144<br>ITALY
1189	CEROM (Comitato Etico della Romagna)<br>Via Moroncelli<br>Meldola, FORLI 47014<br>ITALY
1191	Comitato Etico Campania Nord c/o A.O Azienda Ospedaliera San Giuseppe Moscati di Avellino<br>Contrada Amoretta Citta' Ospedaliera<br>Avellino, 83100<br>ITALY
1264	Comitato Etico<br>Via Elio Chianesi, 53<br>Roma, 00128<br>ITALY

Study Site Number	Independent Ethics Committee or Institutional Review Board Address(es)
1265	Comitato Etico Indipendente<br>Universitaria di Bologna Policlinico S. Orsola Malpighi<br>dell'Azienda Ospedaliero, Via Albertoni, 15<br>Bologna, BO 40138<br>ITALY
1298	Comitato Etico Per Le Attivita' Biomediche "Carlo Romano"<br>via Sergio Pansini 5Comitato Etico Per Le Attivita' Biomediche "Carlo Romano", Universita' Degli Studi Di Napoli "Federico II"<br>Naples, 80131<br>ITALY

JAPAN

Study Site Number	Independent Ethics Committee or Institutional Review Board Address(es)
1323	Aichi Cancer Center Hospital IRB<br>Kanokoden, Chikusa-ku, 1-1<br>Nagoya, AICHI 464-8681<br>JAPAN
1324	National Cancer Center Hospital IRB<br>Tsukiji, Chuo-ku,, 5-1-1<br>Tokyo, 104-0045<br>JAPAN
1325	National Cancer Center Hospital IRB<br>5-1-1 Tsukiji, Chuo-ku<br>Tokyo, 104-0045<br>JAPAN
1326	National Hospital Organization Osaka National Hospital IRB<br>Hoenzaka, Chuo-ku, 2-1-14<br>Osaka, OSAKA 540-0006<br>JAPAN
1327	Hakuaikai Medical Corporation Sagara Hospital Institutional Review Board<br>3-31, Matsubara-cho<br>Kagoshima, KAGOSHIMA 892-0833<br>JAPAN
1328	Iwate Medical University IRB<br>19-1, Uchimaru<br>Morioka-shi, IWATE 020-8505<br>JAPAN
1329	National Hospital Organization Shikoku Cancer Center IRB<br>160, Kou Minamiumemoto-machi<br>Matsuyama, EHIME 791-0280<br>JAPAN

Study Site Number	Independent Ethics Committee or Institutional Review Board Address(es)
1330	National Hospital Organization Kyushu Cancer Center IRB<br>3-1-1,Notame, Minami-ku<br>Fukuoka, FUKUOKA 811-1395<br>JAPAN
1331	National Hospital Organization Hokkaido Cancer Center Institutional Review Board<br>Kikusui, Shiroishi-ku, 4-2-3-54<br>Sapporo-city, HOKKAIDO 003-0804<br>JAPAN
1332	Chiba Cancer Center IRB<br>Nitona-cho, Chuo-ku, 666-2<br>Chiba, CHIBA 260-8717<br>JAPAN
1333	Hiroshima City Hiroshima Citizens Hospital Institutional Review Board<br>Moto-machi, Naka-ku, 7-33<br>Hiroshima, HIROSHIMA 730-8518<br>JAPAN
1334	Kumamoto University Hospital IRB<br>1-1-1, Honjo, Chuo-ku<br>Kumamoto-city, KUMAMOTO 860-8556<br>JAPAN
1335	Saitama Cancer Center IRB<br>Komuro, Ina-cho, 780<br>Kita-adachi-gun, SAITAMA 362-0806<br>JAPAN
1337	Kumamoto City Hospital IRB<br>1-1-60 Koto, Higashi-ku<br>Kumamoto-city, KUMAMOTO 862-8505<br>JAPAN
1340	Niigata Cancer Center Hospital Institutional Review Board<br>2-15-3 Kawagishi-cho, Chuo-Ku<br>Niigata, 951-8566<br>JAPAN

KOREA, REPUBLIC OF

Study Site Number	Independent Ethics Committee or Institutional Review Board Address(es)
1172	Samsung Medical Center IRB<br>Samsung Medical Center<br>81 Irwon-ro, Gangnam-gu<br>Seoul, 06351<br>KOREA, REPUBLIC OF
1173	Seoul National University Hospital Institutional Review Board<br>101, Daehak-ro, Jongno-gu, Seoul National University Hospital Institutional Review Board<br>Seoul, 03080<br>KOREA, REPUBLIC OF
1174	Asan Medical Center IRB<br>Asan Medical Center<br>88, Olympic-ro, 43-Gil, Songpa-Gu<br>Seoul, 05505<br>KOREA, REPUBLIC OF
1175	Seoul National University Bundang Hospital IRB<br>82, Gumi-ro 173beon-gil, Bundang-gu<br>Seongnam-si, GYEONGGI-DO 13620<br>KOREA, REPUBLIC OF
1177	National Cancer Center, Institutional Review Board<br>323 Ilsan-ro, Ilsandong-gu<br>Goyang-si, GYEONGGI-DO 10408<br>KOREA, REPUBLIC OF
1179	Severance Hospital, IRB, Human Research Protection Center<br>50 Yonsei-ro, Seodaemun-gu, Severance Hospital, Yonsei University Health System<br>Seoul, 03722<br>KOREA, REPUBLIC OF

NEW ZEALAND

**Study Site Number**	**Independent Ethics Committee or Institutional Review Board Address(es)**
1169	Health and Disability Ethics Committee (HDEC)<br>No 1 The Terrace, Ministry of Health<br>Wellington, PO Box 5013<br>NEW ZEALAND

NO SCA COUNTRY SPECIFIED

Study Site Number	Independent Ethics Committee or Institutional Review Board Address(es)
1103	Clinical Research Ethics Committee of the Cork Teaching Hospitals<br>6 Little Hanover Street, Lancaster Hall<br>Cork<br>IRELAND

POLAND

Study Site Number	Independent Ethics Committee or Institutional Review Board Address(es)
1052	Niezalezna Komisja Bioetyczna ds. Badan Naukowych przy Gdanskim Uniwersytecie Medycznym (GUMed),<br>M. Sklodowskiej - Curie 3a, Str<br>Gdansk, 80-210<br>POLAND
1053	Niezalezna Komisja Bioetyczna do Spraw Badan Naukowych przy GUMed<br>M. Sklodowskiej Curie 3A, Str.<br>Gdansk, 80-211<br>POLAND
1099	Niezalezna Komisja Bioetyczna do Spraw Badan Naukowych przy GUMed<br>M. Sklodowskiej - Curie 3a<br>Gdansk, 80-211<br>POLAND
1245	Niezalezna Komisja Bioetyczna do Spraw Badan Naukowych przy GUMed<br>M. Sklodowskiej - Curie 3a<br>Gdansk, 80-211<br>POLAND

RUSSIAN FEDERATION

Study Site Number	Independent Ethics Committee or Institutional Review Board Address(es)
1051	Local Ethics Committee<br>Kursk Regional Clinical Oncology Dispensary<br>20, ul Pirogova, Healthcare Committee of Kursk Region<br>Kursk, 305035<br>RUSSIAN FEDERATION<br><br>Ethics Council under the Ministry of Health of the Russian Federation<br>3, Rakhmanovskiy pereulok<br>Moscow, 127994<br>RUSSIAN FEDERATION
1055	LEC of State Budget Healtcare Institution<br>37-39 Liteiny prospect, "Leningrad Region Oncology Dispensary"<br>Saint-Petersburg, 191014<br>RUSSIAN FEDERATION<br><br>Ethics Council under the Ministry of Health of the Russian Federation<br>3, Rakhmanovskiy pereulok<br>Moscow, 127994<br>RUSSIAN FEDERATION
1056	Ethics Council at the Ministry of Health of the Russian Federation<br>3, Rakhmanovskiy pereulok<br>Moscow, 127994<br>RUSSIAN FEDERATION<br><br>LEC of SBMI Republican Clinical Oncology Dispensary under the Ministry of health Care of RB<br>73/1. Oktyabrya pr.<br>Ufa, RUSSIA 450054<br>RUSSIAN FEDERATION
1058	LEC of Saint-Petersburg State Budget Healthcare Institution (SBHCI)<br>City Clinical Oncology Dispensary<br>56, Veteranov prospect<br>Saint-Petersburg, 197022<br>RUSSIAN FEDERATION

Study Site Number	Independent Ethics Committee or Institutional Review Board Address(es)
	Ethics Council of the Ministry of Health of the Russian Federation<br>3 Rakhmanovsky pereulok<br>Moscow, 127994<br>RUSSIAN FEDERATION
1059	LEC of State Budget institution of Healthcare Omsk Regional Clinical Oncology Dispensary<br>9/1, Zavertyaeva, Str.<br>Omsk, 644013<br>RUSSIAN FEDERATION
	Ethics Council of the Ministry of Health of the Russian Federation<br>3 Rakhmanovsky pereulok<br>Moscow, 127994<br>RUSSIAN FEDERATION
1124	Local Ethical Committee<br>State Medicoprophylactic Institution 'Chelyabinsk Regional Oncology Center'<br>42, Blukhera str.<br>Chelyabinsk, 454076<br>RUSSIAN FEDERATION
	Ethics Council of the Ministry of Health of the Russian Federation<br>3 Rakhmanovsky pereulok<br>Moscow, 127994<br>RUSSIAN FEDERATION
1216	Ethics Committee of N.N.Blokhin Cancer Research Center RAMS<br>N.N.Blokhin Cancer Research Center RAMS<br>24, Kashirskoye sh.<br>Moscow, 115478<br>RUSSIAN FEDERATION
	Ethics Council of the Ministry of Health of the Russian Federation<br>3 Rakhmanovsky pereulok<br>Moscow, 127994<br>RUSSIAN FEDERATION

Study Site Number	Independent Ethics Committee or Institutional Review Board Address(es)
1286	LEC based on Non-State HealthCare agency "Road Clinical Hospital of PLC" Russian Railways"<br>27, prospect MechnikovaNon-State Institution of Healthcare, RZHD OJSC RR<br>St. Petersburg, 195271<br>RUSSIAN FEDERATION
	Ethics Council under the Ministry of Health of the Russian Federation<br>3, Rakhmanovskiy pereulok<br>Moscow, 127994<br>RUSSIAN FEDERATION
1287	Expert Board/Ethics Committee of Republic<br>Clinical Oncology Dispensary of Ministry of Health of Republic of Tatarstan<br>29, Sibirsky trakt<br>Kazan, 420029<br>RUSSIAN FEDERATION
	Ethics Council of the Ministry of Health of the Russian Federation<br>3 Rakhmanovsky pereulok<br>Moscow, 127994<br>RUSSIAN FEDERATION
1297	Ethics Committee of State Educational Institution of Higher Professional Education Ryazan State Medical University I.P. Pavlov, 9, Vysokovoltnaya ulitsa<br>Ryazan, 390026<br>RUSSIAN FEDERATION
	Ethics Council at the Ministry of Health of the Russian Federation<br>3, Rakhmanovskij pereulok<br>Moscow, 127994<br>RUSSIAN FEDERATION
1308	EC of SAHI of Moscow "Moscow City Oncology Hospital #62 of Health Department of Moscow"<br>Moscow Healthcare Department<br>Settlement Istra 27 bld 1-26, Krasnogorskiy Disctrict<br>Moscow area, 143423<br>RUSSIAN FEDERATION

**Study Site Number**	**Independent Ethics Committee or Institutional Review Board Address(es)**
	Ethics Council of the Ministry of Health of the Russian Federation<br>3 Rakhmanovsky pereulok<br>Moscow, 127994<br>RUSSIAN FEDERATION

SPAIN

Study Site Number	Independent Ethics Committee or Institutional Review Board Address(es)
1079	Comite etico de investigacion clinica<br>C/Feixa Llarga, s/nHospital universitario de Bellvitge, L'Hospitalet del Llobregat, secretaria administrativa del CEIC. Edifici de recerca<br>Barcelona, 08907<br>SPAIN
1080	Comite Etico de Investigación Clínica. Hospital Universitari de Bellvitge<br>Secretaría Administrativa del CEIC,Edifici Unitat de Recerca.<br>L'Hospitalet de Llobregat, C/ Feixa Llarga, s/n<br>Barcelona, 08907<br>SPAIN
1081	Comite Etico de Investigacion Clinica, Hospital Universitario de Bellvitge<br>Secretaria Administrativa del CEIC, Edifici Unitat de Recerca<br>C/ Feixa Llarga, s/n<br>L'Hospitalet de Llobregat, BARCELONA 08907<br>SPAIN
1082	Comite Etico de Investigacion Clinica, Hospital Universitario de Bellvitge<br>Secretaria Administrativa del CEIC, Edifici Unitat de Recerca<br>C/ Feixa Llarga, s/n<br>L'Hospitalet de Llobregat, BARCELONA 08907<br>SPAIN
	Comite Etico de Investigacion Clinica de Las Islas Baleares<br>Camino de Jesus, 38, Conselleria de Salut i Consum<br>Palma de Mallorca, ISLAS BALEARES 07011<br>SPAIN
1083	Comite Etico de Investigación Clínica. Hospital Universitari de Bellvitge<br>Secretaría Administrativa del CEIC,Edifici Unitat de Recerca.<br>L'Hospitalet de Llobregat, C/ Feixa Llarga, s/n<br>Barcelona, 08907<br>SPAIN

Study Site Number	Independent Ethics Committee or Institutional Review Board Address(es)
1084	Comite Etico de Investigacion Clinica, Hospital Universitario de Bellvitge<br>Secretaria Administrativa del CEIC, Edifici Unitat de Recerca<br>C/ Feixa Llarga, s/n<br>L'Hospitalet de Llobregat, BARCELONA 08907<br>SPAIN
1085	Comite Etico de Investigacion Clinica, Hospital Universitario de Bellvitge<br>Secretaria Administrativa del CEIC, Edifici Unitat de Recerca<br>C/ Feixa Llarga, s/n<br>L'Hospitalet de Llobregat, BARCELONA 08907<br>SPAIN
1086	Comite Etico de Investigacion Clinica, Hospital Universitario de Bellvitge<br>Secretaria Administrativa del CEIC, Edifici Unitat de Recerca<br>C/ Feixa Llarga, s/n<br>L'Hospitalet de Llobregat, BARCELONA 08907<br>SPAIN
1087	Comite Etico de Investigacion Clinica, Hospital Universitario de Bellvitge<br>Secretaria Administrativa del CEIC, Edifici Unitat de Recerca<br>C/ Feixa Llarga, s/n<br>L'Hospitalet de Llobregat, BARCELONA 08907<br>SPAIN
1088	Comite etico de investigacion clinica<br>C/Feixa Llarga, s/nHospital universitario de Bellvitge, L'Hospitalet del Llobregat, secretaria administrativa del CEIC. Edifici de recerca<br>Barcelona, 08907<br>SPAIN
1089	Comite Etico de Investigacion Clinica, Hospital Universitario de Bellvitge<br>Secretaria Administrativa del CEIC, Edifici Unitat de Recerca<br>C/ Feixa Llarga, s/n<br>L'Hospitalet de Llobregat, BARCELONA 08907<br>SPAIN

Study Site Number	Independent Ethics Committee or Institutional Review Board Address(es)
1090	Comite etico de investigacion clinica<br>Edifici Unitat de recerca<br>secretaria administrativa del CEIC.C/Feixa Llarga, s/n, Hospital universitario de Bellvitge L'Hospitalet del Llobregat<br>Barcelona, 08907<br>SPAIN
1091	Comite etico de investigacion clinica<br>C/Feixa Llarga, s/nHospital universitario de Bellvitge, L'Hospitalet del Llobregat, secretaria administrativa del CEIC. Edifici de recerca<br>Barcelona, 08907<br>SPAIN
1092	Comite Etico de Investigacion Clinica<br>Secretaria Administrativa del CEIC, Edifici Unitat de Recerca<br>Hospital Universitario de Bellvitge, C/ Feixa Llarga, s/n<br>L'Hospitalet de Llobregat, BARCELONA 08907<br>SPAIN
1105	Comite Etico de Investigacion Clinica<br>Secretaría Administrativa del CEIC, Edifici Unitat de Recerca.<br>Hospital Universitario de Bellvitge, L'Hospitalet de Llobregat, C/Feixa Llarga, s/n<br>Barcelona, 08907<br>SPAIN
1106	Comite Etico de Investigacion Clinica<br>Secretaría Administrativa del CEIC, Edifici Unitat de Recerca.<br>Hospital Universitario de Bellvitge, L'Hospitalet de Llobregat, C/Feixa Llarga, s/n<br>Barcelona, 08907<br>SPAIN
1107	Comite Etico de Investigacion Clinica<br>Secretaría Administrativa del CEIC, Edifici Unitat de Recerca.<br>Hospital Universitario de Bellvitge, L'Hospitalet de Llobregat, C/Feixa Llarga, s/n<br>Barcelona, 08907<br>SPAIN

Study Site Number	Independent Ethics Committee or Institutional Review Board Address(es)
1116	Comite Etico de Investigacion Clinica<br>Secretaría Administrativa del CEIC, Edifici Unitat de Recerca.<br>Hospital Universitario de Bellvitge, L'Hospitalet de Llobregat, C/Feixa Llarga, s/n<br>Barcelona, 08907<br>SPAIN
1117	Comite Etico de Investigacion Clinica<br>Secretaría Administrativa del CEIC, Edifici Unitat de Recerca.<br>Hospital Universitario de Bellvitge, L'Hospitalet de Llobregat, C/Feixa Llarga, s/n<br>Barcelona, 08907<br>SPAIN
1146	Hospital Fundación de Alcorcón<br>C/ Budapest 1 - Planta SótanoComité Ético de Investigación Clínica - Área 8, Fundación Hospital Alcorcón<br>Madrid, ALCORCÓN 28922<br>SPAIN
	Comite etico de investigacion clinica<br>C/Feixa Llarga, s/nHospital universitario de Bellvitge, L'Hospitalet del Llobregat, secretaria administrativa del CEIC. Edifici de recerca<br>Barcelona, 08907<br>SPAIN
1147	Comite etico de investigacion clinica<br>C/Feixa Llarga, s/nHospital universitario de Bellvitge, L'Hospitalet de Llobregat, secretaria administrativa del CEIC. Edifici de recerca<br>Barcelona, 08907<br>SPAIN
1149	Comite Etico de Investigacion Clinica<br>Secretaría Administrativa del CEIC, Edifici Unitat de Recerca.<br>Hospital Universitario de Bellvitge, L'Hospitalet de Llobregat, C/Feixa Llarga, s/n<br>Barcelona, 08907<br>SPAIN

TAIWAN

Study Site Number	Independent Ethics Committee or Institutional Review Board Address(es)
1260	Institutional Review Board, Koo Foundation Sun Yat-Sen Cancer Center<br>No.125, Lih-Der Road, Taiwan R.O.C<br>Bei-Tou District, TAIPEI 11259<br>TAIWAN
1261	Institutional Review Board, Taipei Veterans General Hospital<br>No.201, Sec. 2, Shipai Rd, Beitou District<br>Taipei, 11217<br>TAIWAN
1266	Mackay Memory Hospital Institutional Review Board<br>No. 92, Section 2, Zhongshan North RoadMACKAY MEMORIAL HOSPITAL Institutional Review Board, Zhongshan District, 8 F<br>Taipei, 10449<br>TAIWAN
1311	National Taiwan University Hospital, Research Ethics Committee<br>Changde St., Zhongzheng Dist., No.1<br>Taipei City, 10002<br>TAIWAN

UKRAINE

Study Site Number	Independent Ethics Committee or Institutional Review Board Address(es)
1143	Ethics Committee of the MI<br>31, Blyzhnya Str., Dnipropetrovsk City Multidisciplinary Clinical Hospital No. 4 of the Dnipropetrovsk City Council<br>Dnipro, 49102<br>UKRAINE
1185	Local Ethic Committee of Lviv State<br>Oncologic Regional Treatment and Diagnostic Center<br>2A, Ja. Gashek St.<br>Lviv, 79031<br>UKRAINE
1186	Local Committee of Ethic Issues of Municipal Institution<br>Local Committee of Ethic Issues of Municipal Institution Zaporizhzhia Regional Clinical Oncology<br>177a, Kultuma Str., Dispensary" ZRA<br>Zaporizhzhia, 69040<br>UKRAINE
1295	Local Ethic Committee of Uzhgorod of Central Municipal Clinical Hospital<br>20, Hryboyedova Str.<br>Uzhgorod, N/A 88000<br>UKRAINE
1300	Local Ethic Committee of MMI "Makiivka City Hospital No.2 of Donetsk Region"<br>12 Ferhanska str<br>Makiivka, N/A 86120<br>UKRAINE
1301	Commission of Ethic Issues of Municipal Non-Profit Enterprise "Regional Centre of Oncology"<br>4, Lisoparkivs'ka Str.<br>Kharkiv, 61070<br>UKRAINE
1304	Local Committee of Ethic Issues of Regional Municipal Establishment<br>Sumy Regional Clinical Oncology Dispensary<br>31, Pryvokzalna Str.<br>Sumy, 40005<br>UKRAINE

UNITED KINGDOM

Study Site Number	Independent Ethics Committee or Institutional Review Board Address(es)
1171	South East Coast-Brighton and Sussex REC<br>80 London Road, Ground Floor, Skipton House<br>London, SE1 6LH<br>UNITED KINGDOM
1249	South East Coast- Brighton and Sussex REC<br>80 London Road Ground Floor, Skipton house<br>London, SE1 6LH<br>UNITED KINGDOM
1250	NRES Committee south East Coast-Brighton and Sussex health Research Authority<br>80 London Road, Ground Floor Skipton House<br>London, SE1 6LH<br>UNITED KINGDOM
1251	South East Coast- Brighton and Sussex REC<br>80 London Road Ground Floor, Skipton house<br>London, SE1 6LH<br>UNITED KINGDOM
1271	NRES Committee London - Central<br>80 London RoadNRES Committee South East Coast - Brighton and Sussex Health Research Authority, Ground Floor, Skipton House<br>London, SE1 6LH<br>UNITED KINGDOM
1310	South East Coast- Brighton and Sussex REC<br>80 London Road Ground Floor, Skipton house<br>London, SE1 6LH<br>UNITED KINGDOM
1320	South East Coast- Brighton and Sussex REC<br>80 London Road Ground Floor, Skipton house<br>London, SE1 6LH<br>UNITED KINGDOM

UNITED STATES

Study Site Number	Independent Ethics Committee or Institutional Review Board Address(es)
1001	Western Institutional Review Board<br>1019 39th Ave SE, Ste 120<br>Puyallup, WA 98374-2115<br>UNITED STATES
1003	Western Institutional Review Board<br>1019 39th Ave SE, Ste 120<br>Puyallup, WA 98374-2115<br>UNITED STATES
1004	Sparrow Institution Research Review Committee Office of Research Oversight and Compliance<br>Sparrow Institutional Review Board, 1215 E Michigan Ave. PO Box 30480<br>Lansing, MI 48090-7890<br>UNITED STATES
1005	Western Institutional Review Board<br>1019 39th Ave SE, Ste 120<br>Puyallup, WA 98374-2115<br>UNITED STATES
1006	Western Institutional Review Board<br>1019 39th Ave SE, Ste 120<br>Puyallup, WA 98374-2115<br>UNITED STATES
1007	Western Institutional Review Board<br>1019 39th Ave SE, Ste 120<br>Puyallup, WA 98374-2115<br>UNITED STATES
1008	Western Institutional Review Board<br>1019 39th Ave SE, Ste 120<br>Puyallup, WA 98374-2115<br>UNITED STATES

Study Site Number	Independent Ethics Committee or Institutional Review Board Address(es)
1009	UCLA - Office of the Human Research Protection Program<br>10889 Wilshire Blvd., Ste. 830<br>Los Angeles, CA 90024<br>UNITED STATES
1010	Western Institutional Review Board<br>1019 39th Ave SE, Ste 120<br>Puyallup, WA 98374-2115<br>UNITED STATES
1011	OHSU Institutional Review Board<br>3181 Sw Sam Jackson Park Rd, L106-RI<br>Portland, OR 97239<br>UNITED STATES
1012	Western Institutional Review Board<br>1019 39th Ave SE, Ste 120<br>Puyallup, WA 98374-2115<br>UNITED STATES
1013	Western Institutional Review Board<br>1019 39th Ave SE, Ste 120<br>Puyallup, WA 98374-2115<br>UNITED STATES
1014	Western Institutional Review Board<br>1019 39th Ave SE, Ste 120<br>Puyallup, WA 98374-2115<br>UNITED STATES
1017	US Oncology Incorporated<br>10101 Woodloch Forest Dr, Institutional Review Board<br>The Woodlands, TX 77380<br>UNITED STATES
1019	Kaiser Permanente Northwest Institutional Review Board<br>3800 N Interstate Ave, Research Subjects Protection Ofc<br>Portland, OR 97227<br>UNITED STATES

Study Site Number	Independent Ethics Committee or Institutional Review Board Address(es)
1020	Western Institutional Review Board<br>1019 39TH AVE SE<br>PUYALLUP, WA 98374<br>UNITED STATES
1023	University of Miami Institutional Review Board<br>1400 NW 10th Ave, Ste 1200A<br>Miami, FL 33136<br>UNITED STATES
1024	Western Institutional Review Board<br>1019 39th Ave SE, Ste 120<br>Puyallup, WA 98374-2115<br>UNITED STATES
1025	Western Institutional Review Board<br>1019 39th Ave SE, Ste 120<br>Puyallup, WA 98374-2115<br>UNITED STATES
1026	Columbia University Medical Center Institutional Review Board<br>154 Haven Ave, First Fl<br>New York, NY 10032<br>UNITED STATES
1028	Human Subject Protection Program Office (HSPPO)<br>MedCenter One<br>501 E Broadway, Ste 200<br>Louisville, KY 40202<br>UNITED STATES
1029	Western Institutional Review Board<br>1019 39th Ave SE, Ste 120<br>Puyallup, WA 98374-2115<br>UNITED STATES

Study Site Number	Independent Ethics Committee or Institutional Review Board Address(es)
1030	West Virginia University Institutional Review Board<br>Chestnut Ridge Research Bldg, 866 Chestnut Ridge Rd, PO Box 6845<br>Morgantown, WV 26506<br>UNITED STATES
1031	University of Southern California Health Science Campus Institutional Review Board<br>LAC/USC Medical Center<br>1200 N State St, General Hospital Ste 4700<br>Los Angeles, CA 90033<br>UNITED STATES
1032	Western Institutional Review Board<br>1019 39th Ave SE, Ste 120<br>Puyallup, WA 98374-2115<br>UNITED STATES
1033	Western Institutional Review Board<br>1019 39th Ave SE, Ste 120<br>Puyallup, WA 98374-2115<br>UNITED STATES
1035	Mercy Hospital St. Louis Institutional Review Board<br>621 S. New Ballas Rd., Ste 6002B<br>St. Louis, MO 63141<br>UNITED STATES
1040	Western Institutional Review Board<br>1019 39th Ave SE, Ste 120<br>Puyallup, WA 98374-2115<br>UNITED STATES
1078	UCSF IRB<br>3333 California St, Ste 315<br>San Francisco, CA 94118<br>UNITED STATES

Study Site Number	Independent Ethics Committee or Institutional Review Board Address(es)
1109	US Oncology Incorporated<br>10101 Woodloch Forest Dr, Institutional Review Board<br>The Woodlands, TX 77380<br>UNITED STATES
1110	US Oncology Incorporated<br>10101 Woodloch Forest Dr, Institutional Review Board<br>The Woodlands, TX 77380<br>UNITED STATES
1111	Chesapeake IRB<br>6940 Columbia Gateway Dr<br>Columbia, MD 21046-3403<br>UNITED STATES
1112	US Oncology Incorporated<br>10101 Woodloch Forest Dr, Institutional Review Board<br>The Woodlands, TX 77380<br>UNITED STATES
1125	US Oncology Incorporated<br>10101 Woodloch Forest Dr, Institutional Review Board<br>The Woodlands, TX 77380<br>UNITED STATES
1126	US Oncology Incorporated<br>10101 Woodloch Forest Dr, Institutional Review Board<br>The Woodlands, TX 77380<br>UNITED STATES
1127	Western Institutional Review Board<br>1019 39th Ave SE, Ste 120<br>Puyallup, WA 98374-2115<br>UNITED STATES
1128	US Oncology Incorporated<br>10101 Woodloch Forest Dr, Institutional Review Board<br>The Woodlands, TX 77380<br>UNITED STATES

Study Site Number	Independent Ethics Committee or Institutional Review Board Address(es)
1129	Yale University Institutional Review Board #2, 3, 4B, 5<br>150 Munson StreetHuman Investigation Committee I, II, III, IV, 3rd Fl<br>New Haven, CT 06520<br>UNITED STATES
1131	Western Institutional Review Board<br>1019 39th Ave SE, Ste 120<br>Puyallup, WA 98374-2115<br>UNITED STATES
1132	US Oncology Incorporated<br>10101 Woodloch Forest Dr, Institutional Review Board<br>The Woodlands, TX 77380<br>UNITED STATES
1133	University of Michigan Review Board for Human Subject Research (IRBMED)<br>2800 Plymouth Rd<br>Ann Arbor, MI 48109<br>UNITED STATES
1134	Department of Research Programs, Walter Reed National Military Medical Center IRB<br>8901 Wiscosin AvenueBuilding 17, Rm 3055<br>Bethesda, MD 20889-5600<br>UNITED STATES
1162	UT MD Anderson Cancer Center Institutional Review Board<br>7007 Bertner Ave., Unit 1637<br>Houston, TX 77030<br>UNITED STATES
1198	Western Institutional Review Board<br>1019 39th Ave SE, Ste 120<br>Puyallup, WA 98374-2115<br>UNITED STATES
1201	Human Research Protections Office<br>UMB BioPark OneUniversity of Maryland School of Medicine, 800 W Baltimore St, Ste 100<br>Baltimore, MD 21201<br>UNITED STATES

Study Site Number	Independent Ethics Committee or Institutional Review Board Address(es)
1203	US Oncology Incorporated<br>10101 Woodloch Forest Dr, Institutional Review Board<br>The Woodlands, TX 77380<br>UNITED STATES
1213	HealthPartners Institute Institutional Review Board<br>3311 E. Old Shakopee Rd.<br>Bloomington, MN 55425<br>UNITED STATES
1214	Western Institutional Review Board<br>1019 39th Ave SE, Ste 120<br>Puyallup, WA 98374-2115<br>UNITED STATES
1230	Western Institutional Review Board<br>1019 39th Ave SE, Ste 120<br>Puyallup, WA 98374-2115<br>UNITED STATES
1235	Kaiser Permanente Southern California Institutional Review Board<br>393 E Walnut St, 2nd Fl<br>Pasadena, CA 91188<br>UNITED STATES
1246	Western Institutional Review Board<br>1019 39th Ave SE, Ste 120<br>Puyallup, WA 98374-2115<br>UNITED STATES
1280	Western Institutional Review Board<br>1019 39th Ave SE, Ste 120<br>Puyallup, WA 98374-2115<br>UNITED STATES
1281	Norwalk Hospital<br>34 Maple St, Institutional Review Board<br>Norwalk, CT 06856<br>UNITED STATES

Study Site Number	Independent Ethics Committee or Institutional Review Board Address(es)
1315	US Oncology Incorporated<br>10101 Woodloch Forest Dr, Institutional Review Board<br>The Woodlands, TX 77380<br>UNITED STATES
1316	MedStar Health Research Institute<br>Sw 104 Medical Dental Bldg, 3900 Reservoir Rd, Nw, Georgetown University Oncology Review Board<br>Washington, DC 20057-2197<br>UNITED STATES
1350	Stanford Research Compliance Office<br>1501 S. California Ave.<br>Palo Alto, CA 94304<br>UNITED STATES
1358	Western Institutional Review Board<br>1019 39th Ave SE, Ste 120<br>Puyallup, WA 98374-2115<br>UNITED STATES
1359	Western Institutional Review Board<br>1019 39th Ave SE, Ste 120<br>Puyallup, WA 98374-2115<br>UNITED STATES
1362	Western Institutional Review Board<br>1019 39th Ave SE, Ste 120<br>Puyallup, WA 98374-2115<br>UNITED STATES
1364	Western Institutional Review Board<br>1019 39 th Ave Se, Ste 120<br>Puyallup, WA 98374<br>UNITED STATES
1365	Western Institutional Review Board<br>1019 39 th Ave Se, Ste 120<br>Puyallup, WA 98374<br>UNITED STATES

Study Site Number	Independent Ethics Committee or Institutional Review Board Address(es)
1372	Western Institutional Review Board<br>1019 39th Ave SE, Ste 120<br>Puyallup, WA 98374<br>UNITED STATES
1373	Western Institutional Review Board<br>1019 39th Ave SE, Ste 120<br>Puyallup, WA 98374-2115<br>UNITED STATES
1378	Western Institutional Review Board<br>1019 39th Ave., SE, Ste 120<br>Puyallup, WA 98374<br>UNITED STATES
1387	Western Institutional Review Board<br>1019 39 th Ave Se, Ste 120<br>Puyallup, WA 98374<br>UNITED STATES

Figure S1. CONSORT Diagram of PALOMA-2 study

[a]Patients who discontinued palbociclib or placebo could continue to receive letrozole alone

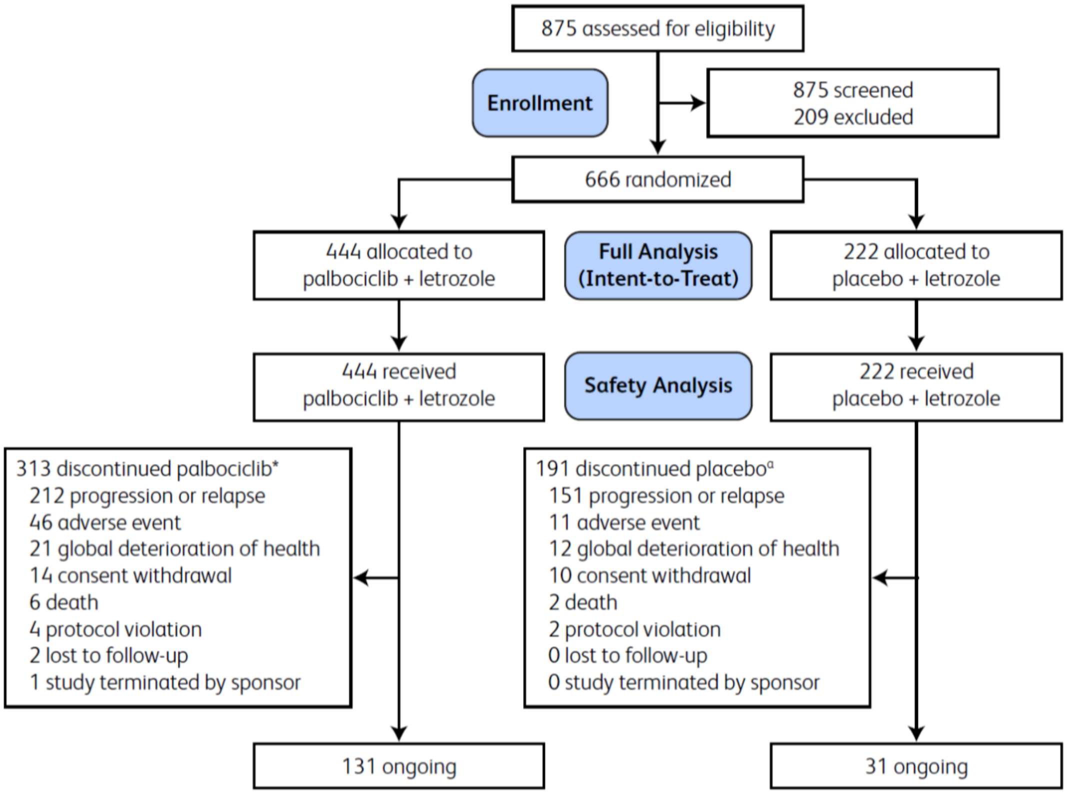

Table S2. Baseline demographic and disease characteristics (intent-to-treat population)

	**Palbociclib + Letrozole (n=444)**	**Placebo + Letrozole (n=222)**
Age, y		
<65	263 (59.2)	141 (63.5)
≥65	181 (40.8)	81 (36.5)
Median (range)	62.0 (30–89)	61.0 (28–88)
Mean (SD)	61.7 (10.6)	60.6 (11.2)
Race		
White	344 (77.5)	172 (77.5)
Asian	65 (14.6)	30 (13.5)
Black	8 (1.8)	3 (1.4)
Other/missing	27 (6.1)	17 (7.7)
ECOG PS		
0	257 (57.9)	102 (45.9)
1	178 (40.1)	117 (52.7)
2	9 (2.0)	2 (1.4)
Stage of disease at initial diagnosis		
I−III	261 (58.8)	137 (61.7)
IV	138 (31.1)	72 (32.4)
Unknown, other, or missing	45 (10.1)	13 (5.9)
Disease site		
Visceral	214 (48.2)	110 (49.5)
Nonvisceral	230 (51.8)	112 (50.5)
Bone only	103 (23.2)	48 (21.6)

Number of disease sites		
1	138 (31.1)	66 (29.7)
2	117 (26.4)	52 (23.4)
3	112 (25.2)	61 (27.5)
4	51 (11.5)	28 (12.6)
>4	26 (5.9)	15 (6.8)
Recurrence type		
Locoregional	2 (0.5)	2 (0.9)
Local	6 (1.4)	3 (1.4)
Regional	3 (0.7)	1 (0.5)
Distant	294 (66.2)	145 (65.3)
Newly diagnosed	139 (31.3)	71 (32.0)
Treatment-free interval[a]		
De novo metastatic	167 (37.6)	81 (36.5)
≤12 mo	98 (22.1)	48 (21.6)
>12 mo	179 (40.3)	93 (41.9)
Prior (neo)adjuvant therapies		
Chemotherapeutic	213 (48.0)	109 (49.1)
Neoadjuvant	54 (12.2)	32 (14.4)
Adjuvant	180 (40.5)	89 (40.1)
Adjuvant endocrine therapy[b]	250 (56.3)	126 (56.8)

Abbreviations: *ECOG PS* Eastern Cooperative Oncology Group performance status

Values are n (%) unless otherwise noted

[a] Defined as the time from (neo)adjuvant therapy to recurrence; percentage calculated based on number of patients who received (neo)adjuvant therapy

[b] Patients who progressed while on or within 12 months from completion of prior anastrozole or letrozole were excluded

Table S3. Exposure to palbociclib or placebo (as-treated population)[a]

	Palbociclib + Letrozole<br>n=444	Placebo + Letrozole<br>n=222
Number of cycles, n	22.9 (14.6)	18.7 (14.5)
Duration of treatments,[b] months,		
Mean	21.9 (14.1)	17.0 (13.4)
Median	20.1 (0.03–49.0)	13.6 (0.33–50.4)
Average daily dose administered,[c] mg		
Mean	115.9 (13.9)	124.8 (2.1)
Median	125.0 (76.0–125.2)	125.0 (104.7–125.6)
Relative dose,[d] %		
Mean	90.3 (12.6)	99.0 (3.7)
Median	97.7 (57.2–109.5)	100.0 (69.0–102.4)
Patients with ≥1 dose reduction,[e] n (%)	175 (39.4)	4 (1.8)
Patients with a dose interruption,[f] n (%)	309 (69.6)	97 (43.7)
Number of interruptions per patient, n	2.0 (1.0–35.0)	2.0 (1.0–15.0)
Average duration of dose interruption,[g] days/interruption	4.0 (1.0–16.0)	1.0 (1.0–16.0)
Patients with cycle delays,[h] n (%)	315 (70.9)	70 (31.5)
Average duration of cycle delay, days	5 (1.0–82.0)	4.0 (1.0–33.0)

[a] Data represent the mean (standard deviation) or the median (minimum–maximum), unless otherwise indicated

[b] Duration of treatments is defined as the total number of dosing days from first to and including last day of each study treatment

[c] Average daily dose administered = (total dose administered)/(total days on drug)

[d] Relative dose = [(actual total dose)/(intended total dose)]*100%

[e] Dose reduction is any dose reduction from the initial prescribed dose, regardless of its duration; dose interruptions are not counted as reduction

[f] Interruptions include missed dose collected on the case report form and dose with 0 mg administered

[g] Average duration of dose interruptions/cycle delays is the sum of interruption/cycle delay duration divided by the total number of interruptions/delays

[h] Cycle delay is defined as any delay of the cycle start beyond 31 days for any given cycle

Figure S2. Investigator-assessed PFS by baseline TFI of > 2 years (A), >5 years (B), and >10 years (C)

Abbreviations: *NE* not estimable, *NR* not reached, *PFS* progression-free survival

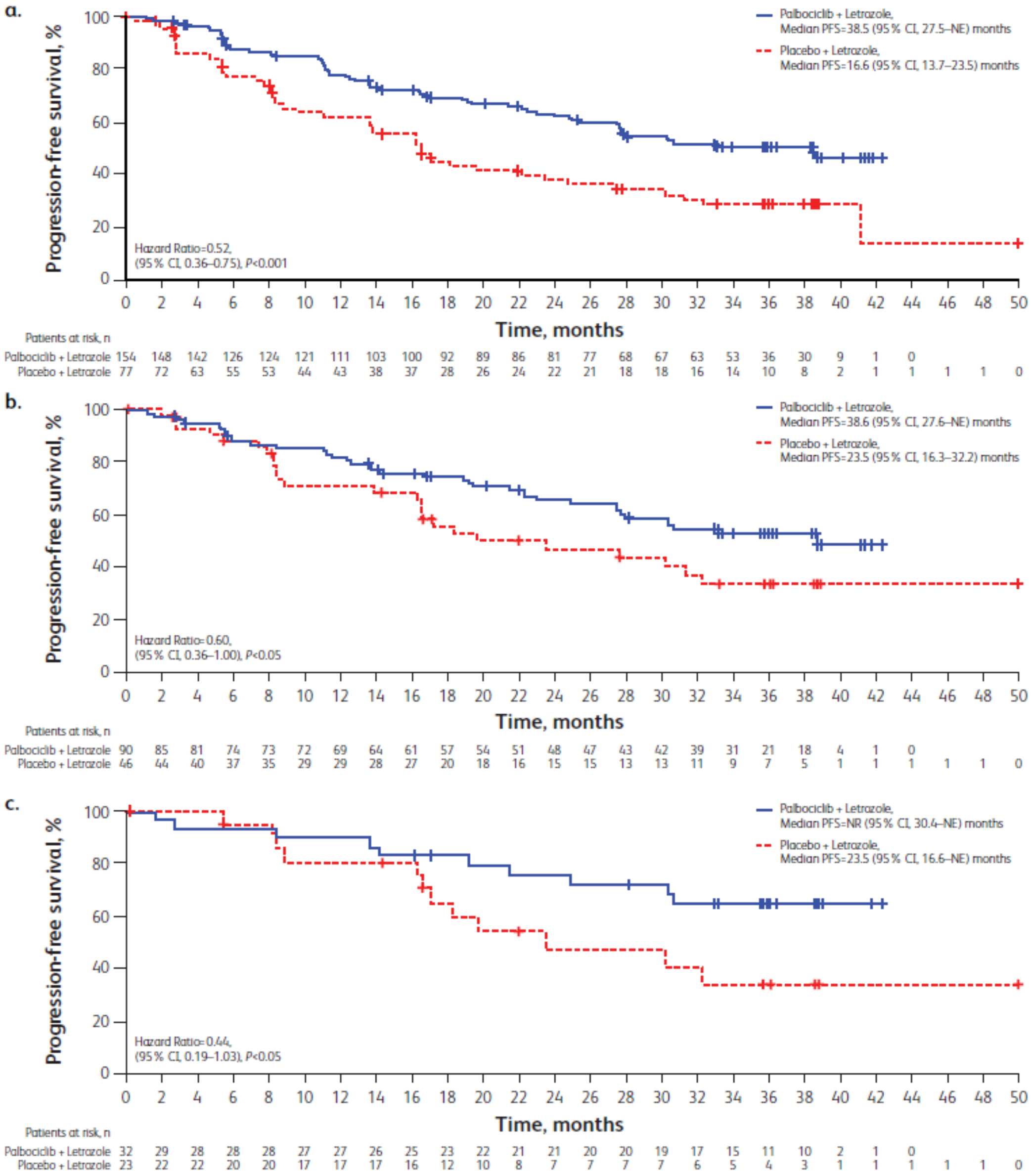

Supplementary Text

Time to Second Subsequent Therapy

Of the 138 and 97 patients in the palbociclib-letrozole and placebo-letrozole arms, respectively, who received a second subsequent therapy after discontinuing study treatment, chemotherapy was the most common treatment (63.0% and 48.5%); 36.2% and 48.5% received endocrine therapy (most commonly fulvestrant and exemestane), and 10.9% and 7.2% received everolimus (**Table S4)**. A CDK4/6 inhibitor was received as the second subsequent therapy postprogression by 0.7% patients in the palbociclib-letrozole arm and by 10.3% of patients in the placebo-letrozole arm; another investigational drug was received by 5.1% and 5.2% of patients, respectively **(Table S4)**.

Figure S3. STEPP analyses. (**a)** All patients who had received adjuvant treatment. **(b)** Patients also with visceral disease. (**c)** Patients also with nonvisceral disease

Abbreviations: *TFI* treatment-free interval, *PFS* progression-free survival, *STEPP* subpopulation treatment effect pattern plot

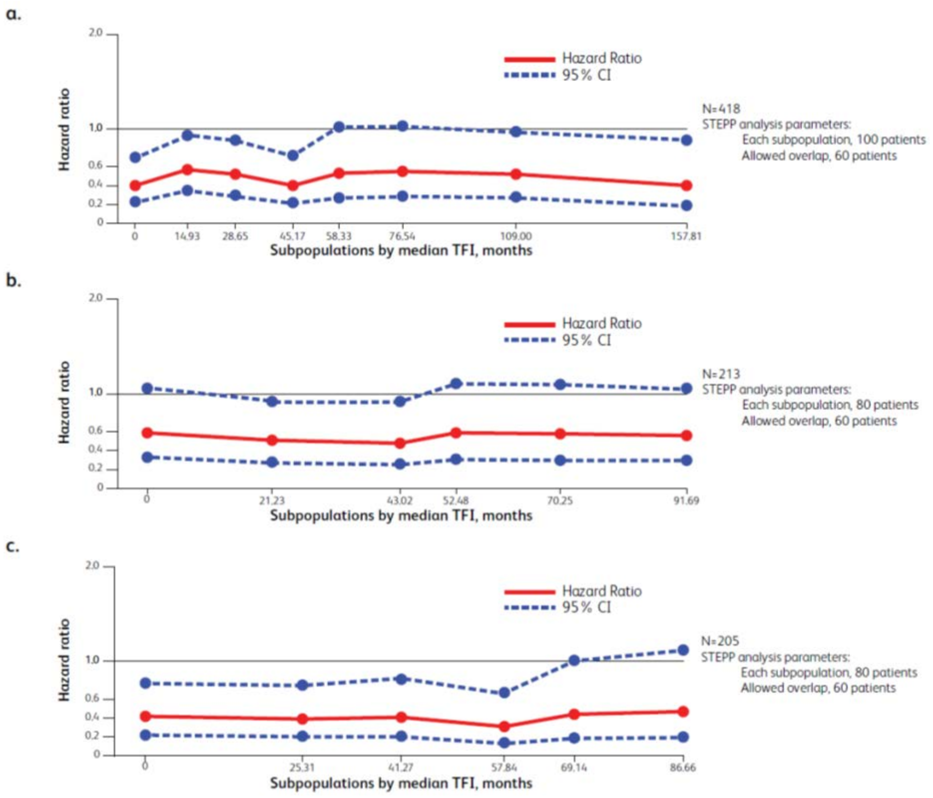

Table S4. Summary of first subsequent systemic anticancer therapies

Systemic Anticancer Therapy	Palbociclib + Letrozole (*n*=444)	Placebo + Letrozole (*n*=222)
**First subsequent therapy, *n* (%)**[a,b]	227 (51.1)	150 (67.6)
Endocrine therapy	138 (60.8)	87 (58.0)
Selective estrogen receptor degraders (fulvestrant)	70 (30.8)	44 (29.3)
Steroidal aromatase inhibitors (exemestane)	49 (21.6)	35 (23.3)
Selective estrogen receptor modulators	16 (7.1)	5 (3.3)
Nonsteroidal aromatase inhibitors	3 (1.3)	3 (2.0)
CDK inhibitor	0	13 (8.7)
CDK4/6 inhibitor (palbociclib)	0	13 (8.7)
Chemotherapy	83 (36.6)	51 (34.0)
Antimetabolites	35 (15.4)	23 (15.3)
Capecitabine	28 (12.3)	19 (12.7)
Fluorouracil	3 (1.3)	1 (0.7)
Gemcitabine	2 (0.9)	1 (0.7)
Gimeracil with oteracil potassium/tegafur	1 (0.4)	0
Methotrexate	1 (0.4)	1 (0.7)
Tegafur	0	1 (0.7)

Taxanes	33 (14.5)	23 (15.3)
Docetaxel	4 (1.8)	4 (2.7)
Paclitaxel (albumin)	29 (12.8)	19 (12.7)
Anthracyclines	14 (6.2)	7 (4.7)
Alkylating agents	8 (3.5)	7 (4.7)
Vinca alkaloids	3 (1.3)	2 (1.3)
mTOR inhibitor (everolimus)	31 (13.7)	26 (17.3)
Investigational drug	23 (10.1)	17 (11.3)
Angiogenesis inhibitors	2 (0.9)	0
Lucitanib	1 (0.4)	0
Pazopanib	1 (0.4)	0
Aurora A kinase inhibitor (alisertib)	1 (0.4)	0
Bcl-2 inhibitor (venetoclax)	1 (0.4)	0
Gamma secretase inhibitor (nirogacestat)	1 (0.4)	0
Histone deacylate inhibitor (entinostat)	1 (0.4)	0
Investigational drug	14 (6.2)	17 (11.3)
Blinded therapy	9 (4.0)	11 (7.3)
Investigational drug	5 (2.2)	6 (4.0)
Placebo	0	1 (0.7)
mTOR kinase inhibitor (AZD2014)	1 (0.4)	0

PARP inhibitors (veliparib)	1 (0.4)	0
PI3K inhibitors	1 (0.4)	1 (0.7)
BKM120	0	1 (0.7)
Taselisib	1 (0.4)	0

Abbreviations: *Bcl-2,* B-cell lymphoma 2, *CDK* cyclin-dependent kinase, *mTOR* mechanistic target of rapamycin, *PARP* Poly (ADP-ribose) polymerase, *PI3K* phosphoinositide 3-kinase

[a] Percentages are calculated using n (for first subsequent therapy) as the denominator

[b] Single agents listed for a drug class appear in parenthesis

Table S5. Summary of second subsequent systemic anticancer therapies

Systemic Anticancer Therapy	Palbociclib + Letrozole (n=444)	Placebo + Letrozole (n=222)
**Second subsequent therapy, n (%)[a,b]**	138 (31.1)	97 (43.7)
Endocrine therapy	50 (36.2)	47 (48.5)
Selective estrogen receptor degrader (fulvestrant)	23 (16.7)	17 (17.5)
Steroidal aromatase inhibitor (exemestane)	19 (13.8)	18 (18.6)
Selective estrogen receptor modulator	7 (5.1)	7 (7.2)
Nonsteroidal aromatase inhibitor	1 (0.7)	5 (5.2)
CDK inhibitor	1 (0.7)	11 (11.3)
CDK4/6 inhibitor (palbociclib)	1 (0.7)	10 (10.3)
Other CDK inhibitor	0	1 (1.0)
Chemotherapy	87 (63.0)	47 (48.5)
Antimetabolites	45 (32.6)	28 (28.9)
Capecitabine	32 (23.2)	25 (25.8)
Cytarabine	2 (1.5)	0
Fluorouracil	4 (2.9)	1 (1.0)
Gemcitabine	4 (2.9)	1 (1.0)
Gimeracil with oteracil potassium/tegafur	1 (0.7)	0
Methotrexate	3 (2.2)	0

Tegafur	0	1 (1.0)
Taxanes	23 (16.7)	12 (12.4)
Docetaxel	4 (2.9)	0
Paclitaxel (albumin)	19 (13.8)	12 (12.4)
Anthracyclines	12 (8.7)	5 (5.2)
Alkylating agents	10 (7.3)	3 (3.1)
Vinca alkaloid	7 (5.1)	1 (1.0)
mTOR inhibitor (everolimus)	15 (10.9)	7 (7.2)
Investigational drug	7 (5.1)	5 (5.2)
Angiogenesis inhibitor (lucitanib)	1 (0.7)	0
Aurora A kinase inhibitor (alisertib)	0	2 (2.1)
Investigational drug	4 (2.9)	1 (1.0)
Blinded therapy	1 (0.7)	0
Investigational drug	3 (2.2)	1 (1.0)
mTOR kinase inhibitor (AZD2014)	1 (0.7)	0
PI3K inhibitors	1 (0.7)	1 (1.0)
BKM120	1 (0.7)	0
Buparlisib	0	1 (1.0)
PI3K/mTOR inhibitor (gedatolisib)	0	1 (1.0)

Abbreviations: *CDK* cyclin-dependent kinase, *mTOR* mechanistic target of rapamycin, *PI3K* phosphoinositide 3-kinase

[a]Percentages are calculated using n (for second subsequent therapy) as the denominator

[b]Single agents listed for a drug class appear in parenthesis

Table S6 Treatment-emergent AEs of any cause reported in >10% of patients in either arm (safety population)

	Palbociclib + Letrozole n=444				Placebo + Letrozole n=222			
	Grade				Grade			
	Any	3	4	5	Any	3	4	5
All AEs, n (%)[a]	440 (99.1)	284 (64.0)	68 (15.3)	12 (2.7)[d]	213 (95.9)	57 (25.7)	6 (2.7)	4 (1.8)[d]
Hematologic AEs[b,]								
Neutropenia[c]	363 (81.8)	255 (57.4)	52 (11.7)	0	14 (6.3)	2 (0.9)	1 (0.5)	0
Leukopenia	179 (40.3)	108 (24.3)	4 (0.9)	0	5 (2.3)	0	0	0
Anemia	117 (26.4)	25 (5.6)	1 (0.2)	0	21 (9.5)	4 (1.8)	0	0
Thrombocytopenia	87 (19.6)	6 (1.4)	1 (0.2)	0	3 (1.4)	0	0	0
Nonhematologic AEs								
Infections[b]	278 (62.6)	29 (6.5)	4 (0.9)	1 (0.2)[‡]	100 (45.0)	7 (3.2)	0	3 (1.4)
Fatigue	176 (39.6)	10 (2.3)	0	0	63 (28.4)	3 (1.4)	0	0
Arthralgia	167 (37.6)	4 (0.9)	—	—	80 (36.0)	2 (0.9)	—	—
Nausea	165 (37.2)	1 (0.2)	0	0	60 (27.0)	4 (1.8)	0	0
Alopecia	149 (33.6)	—	—	—	36 (16.2)	—	—	—
Stomatitis[b]	140 (31.5)	5 (1.1)	0	0	33 (14.9)	0	0	0
Diarrhea	126 (28.4)	6 (1.4)	0	0	47 (21.2)	3 (1.4)	0	0
Cough	118 (26.6)	1 (0.2)	—	—	45 (20.3)	0	—	—

Back pain	104 (23.4)	7 (1.6)	—	—	50 (22.5)	0	—	—
Headache	103 (23.2)	2 (0.5)	—	—	60 (27.0)	4 (1.8)	—	—
Constipation	96 (21.6)	2 (0.5)	0	0	35 (15.8)	1 (0.5)	0	0
Hot flush	96 (21.6)	0	—	—	69 (31.1)	0	—	—
Rash[b]	88 (19.8)	4 (0.9)	0	0	28 (12.6)	1 (0.5)	0	0
Pain in extremity	82 (18.5)	1 (0.2)	—	—	41 (18.5)	3 (1.4)	—	—
Asthenia	80 (18.0)	12 (2.7)	—	—	27 (12.2)	0	—	—
Decreased appetite	75 (16.9)	3 (0.7)	0	0	20 (9.0)	0	0	0
Vomiting	75 (16.9)	4 (0.9)	0	0	38 (17.1)	3 (1.4)	0	0
Dyspnea	73 (16.4)	6 (1.4)	0	0	34 (15.3)	4 (1.8)	0	0
Dizziness	71 (16.0)	2 (0.5)	—	—	34 (15.3)	0	—	—
Insomnia	69 (15.5)	0	—	—	28 (12.6)	0	—	—
Myalgia	63 (14.2)	1 (0.2)	—	—	20 (9.0)	0	—	—
Abdominal pain	59 (13.3)	6 (1.4)	—	—	15 (6.8)	0	—	—
Dry skin	59 (13.3)	0	—	—	16 (7.2)	0	—	—
Pyrexia	59 (13.3)	0	0	0	20 (9.0)	0	0	0
Peripheral edema	57 (12.8)	0	—	—	15 (6.8)	0	—	—
ALT increased	56 (12.6)	11 (2.5)	1 (0.2)	—	13 (5.9)	0	0	—
AST increased	53 (11.9)	13 (2.9)	0	—	13 (5.9)	2 (0.9)	0	—
Fall	50 (11.3)	3 (0.7)	—	—	15 (6.8)	0	—	—

Dyspepsia	48 (10.8)	1 (0.2)	—	—	27 (12.2)	1 (0.5)	—	—
Musculoskeletal pain	47 (10.6)	1 (0.2)	—	—	19 (8.6)	0	—	—
Dysgeusia	46 (10.4)	—	—	—	11 (5.0)	—	—	—

Abbreviations: *AE* adverse event; *ALT* alanine aminotransferase; *AST* aspartate aminotransferase, *MedDRA* Medical Dictionary for Regulatory Activities, *PT* preferred term, *URTI* upper respiratory tract infection, *UTI* urinary tract infection

[a] All AEs regardless of suspected causal relationship to the study medication; MedDRA (v20.0) coding dictionary applied

[b] Clustered PTs (any event having a PT that is equal to those listed): anemia includes the PTs anemia, hematocrit decreased, and hemoglobin decreased; infections includes any PT under the system organ class infections and infestations; leukopenia includes leukopenia and white blood cell count decreased; neutropenia includes neutropenia and neutrophil count decreased; rash includes dermatitis, dermatitis acneiform, rash, rash erythematous, rash maculopapular, rash popular, rash pruritic, and toxic skin eruption; stomatitis includes aphthous stomatitis, cheilitis, glossitis, glossodynia, mouth ulceration, mucosal inflammation, oral pain, oropharyngeal discomfort, oropharyngeal pain, and stomatitis; thrombocytopenia includes platelet count decreased and thrombocytopenia

[c] In the palbociclib-letrozole arm, grade 3 and 4 febrile neutropenia were reported in 7 (1.6%) and 1 (0.2%) patients, respectively, at the time of the primary analysis (data cutoff date February 26, 2016) and in 7 (1.6%) and 2 (0.5%) patients after the additional 15 months of follow-up (data cutoff date May 31, 2017). The later data cutoff date included an additional 1 (0.2%) patient who developed grade 4 febrile neutropenia with a very slight fever (38.2 ºC) 1 week after the study drug was permanently discontinued. Because the onset of grade 3 or 4 febrile neutropenia takes a median 28 days to emerge after palbociclib is stopped and residual neutropenia is not uncommon for chemotherapy (nab-paclitaxel was the subsequent line of treatment) 7 days after its discontinuation, the chemotherapy appears to contribute to this SAE.

[d] Grade 5 AEs in the palbociclib-letrozole arm were disease progression (n=3), infection (pneumonia) and respiratory failure, pulmonary embolism, acute myocardial infarction, breast cancer, breast cancer metastatic, cardiogenic shock, cardiopulmonary failure, cardiovascular insufficiency and death (all n=1):

note, 1 patient had grade 5 pneumonia (infection) and grade 5 respiratory failure; grade 5 AEs in the placebo-letrozole arm were infections (n=3), pulmonary embolism, and cardiac arrest (both n=1); note: grade 5 pulmonary embolism and grade 5 lower respiratory tract infection were reported in 1 patient.
